# Barcoded Consortium Infections Resolve Cell Type-Dependent Salmonella enterica Serovar Typhimurium Entry Mechanisms

**DOI:** 10.1128/mBio.00603-19

**Published:** 2019-05-21

**Authors:** Maria Letizia Di Martino, Viktor Ek, Wolf-Dietrich Hardt, Jens Eriksson, Mikael E. Sellin

**Affiliations:** a Science for Life Laboratory, Department of Medical Biochemistry and Microbiology, Uppsala University, Uppsala, Sweden; b Institute for Microbiology, ETH Zürich, Zürich, Switzerland; University of Washington

**Keywords:** *Salmonella*, bacterial invasion, genome barcoding, epithelial cells, macrophages, monocytes

## Abstract

Bacterial host cell invasion mechanisms depend on the bacterium’s virulence factors and the properties of the target cell. The enteropathogen Salmonella enterica serovar Typhimurium (*S*.Tm) invades epithelial cell types in the gut mucosa and a variety of immune cell types at later infection stages. The molecular mechanism(s) of host cell entry has, however, been studied predominantly in epithelial cell lines. *S*.Tm uses a type three secretion system (TTSS-1) to translocate effectors into the host cell cytosol, thereby sparking actin ruffle-dependent entry. The ruffles also fuel cooperative invasion by bystander bacteria. In addition, several TTSS-1-independent entry mechanisms exist, involving alternative *S*.Tm virulence factors, or the passive uptake of bacteria by phagocytosis. However, it remains ill-defined how *S*.Tm invasion mechanisms vary between host cells. Here, we developed an internally controlled and scalable method to map *S*.Tm invasion mechanisms across host cell types and conditions. The method relies on host cell infections with consortia of chromosomally tagged wild-type and mutant *S*.Tm strains, where the abundance of each strain can be quantified by qPCR or amplicon sequencing. Using this methodology, we quantified cooccurring TTSS-1-dependent, cooperative, and TTSS-1-independent invasion events in epithelial, monocyte, and macrophage cells. We found *S*.Tm invasion of epithelial cells and monocytes to proceed by a similar MOI-dependent mix of TTSS-1-dependent and cooperative mechanisms. TTSS-1-independent entry was more frequent in macrophages. Still, TTSS-1-dependent invasion dominated during the first minutes of interaction also with this cell type. Finally, the combined action of the SopB/SopE/SopE2 effectors was sufficient to explain TTSS-1-dependent invasion across both epithelial and phagocytic cells.

## Abstract (precis)

Salmonella enterica serovar Typhimurium (*S*.Tm) is a widespread and broad-host-spectrum enteropathogen with the capacity to invade diverse cell types. Still, the molecular basis for the host cell invasion process has largely been inferred from studies of a few selected cell lines. Our work resolves the mechanisms that *Salmonellae* employ to invade prototypical host cell types, i.e., human epithelial, monocyte, and macrophage cells, at a previously unattainable level of temporal and quantitative precision. This highlights efficient bacterium-driven entry into innate immune cells and uncovers a type III secretion system effector module that dominates active bacterial invasion of not only epithelial cells but also monocytes and macrophages. The results are derived from a generalizable method, where we combine barcoding of the bacterial chromosome with mixed consortium infections of cultured host cells. The application of this methodology across bacterial species and infection models will provide a scalable means to address host-pathogen interactions in diverse contexts.

## INTRODUCTION

Enterobacterial pathogens colonize the intestinal lumen and attack the mucosal epithelium, thereby eliciting gut inflammation and diarrheal symptoms. Salmonella enterica subspecies 1 serovar Typhimurium (*S*.Tm) is a prototype enterobacterium for studies of the microbe-host cell interactions that explain disease development. *S*.Tm uses a multitude of virulence factors, e.g., flagella, adhesins, two type III secretion systems (TTSS-1 and TTSS-2), and cognate effectors, to compete in the gut lumen and subvert host tissue responses (1–3). Central to the pathogenicity of *S*.Tm is the bacterium’s abilities to invade host cells and to survive in the intracellular niche. At the gut mucosal surface, *S*.Tm targets epithelial cell types, including M cells, absorptive epithelial cells, and goblet cells (4–8). Bacteria that breach the epithelial barrier can enter monocytes, macrophages, dendritic cells, neutrophils, and lymphocytes present in the underlying lamina propria or systemic organs (9–11). The mucosal inflammatory response also causes influx of activated innate immune cells into the *S*.Tm-filled gut lumen (12, 13). Hence, *S*.Tm encounters, and can invade, a wide variety of host cell types during the infection cycle.

Mechanistic studies of *S*.Tm host cell invasion have predominantly been conducted with cultured epithelial cell lines, e.g., HeLa. This work has given rise to a detailed biochemical model of the entry process (2). *S*.Tm employs TTSS-1 to translocate effectors across the host cell membrane. Five of these effectors impact the host cell actin cytoskeleton. SipA works in concert with the TTSS-1 translocon component SipC to drive actin nucleation (14) and further stabilizes and protects actin filaments from the depolymerizing activities of, e.g., ADF/cofilin and Gelsolin (15, 16). SopB is a lipid phosphatase that alters the phosphatidylinositol-phosphate composition of the plasma membrane inner leaflet, thereby indirectly recruiting cellular Rho GTPases (17–19). SopE and SopE2 function as guanine nucleotide exchange factors (GEFs) that directly activate Rho GTPases, including Cdc42 and Rac1 (20, 21). This activation can be reversed by the effector SptP (22, 23). The combined actions of the TTSS-1 effectors result in transient activation of the WAVE regulatory complex, Arp2/3-dependent actin polymerization, and formation of expansive membrane ruffles that drive entry (2). The *S*.Tm-induced ruffles also fuel a second “cooperative” invasion mechanism—the macropinocytic uptake of proximally located bystander bacteria (24, 25).

While TTSS-1-dependent *S*.Tm invasion of epithelial cell lines has been mapped in some detail, it remains less clear whether the findings can be extrapolated across host cell types. A myosin-II-dependent contractility pathway significantly impacts TTSS-1- and SopB-mediated *S*.Tm invasion of fibroblasts (26). Fibroblasts are, however, unlikely targets for *S*.Tm invasion *in vivo*. The Rck and PagN outer membrane proteins may also under certain conditions promote TTSS-1-independent *Salmonella* invasion (27–29), but the physiological impact of these mechanisms remain poorly understood. Finally, macrophages and dendritic cells can take up both TTSS-1-expressing and nonexpressing *S*.Tm through phagocytosis, but the impact of TTSS-1 expression appears variable (30–32). Systematic studies of the contribution of TTSS-1 effectors during invasion of phagocytes and their progenitors are so far missing.

Bacterial host cell invasion has typically been studied by gentamicin protection assays. This involves parallel infections of cultured host cells with strains of interest, killing of extracellular bacteria with gentamicin, and subsequent plating of intracellular bacteria on selective agar. Such assays are laborious, exhibit limited precision, and are not easily scalable. Some larger-scale methods have been developed recently (33–35), but these are still subject to the inherent experimental noise that stems from well-to-well, plate-to-plate, or batch-to-batch variation.

Here, we have developed a single-well, internally controlled, infection method to resolve and quantify *S*.Tm invasion mechanisms in distinct host cells at high temporal resolution. The method relies on infection of cultured host cells with barcoded consortia containing multiple (wild-type or mutant) *S*.Tm strains. Each strain can be recognized by a unique genetic tag located at an inert location on the *Salmonella* chromosome (36). This permits quantification of the relative abundance of each strain in the inoculum and the intracellular population by quantitative PCR (qPCR) or amplicon sequencing (Amplicon Seq). We have by this approach quantified cooccurring TTSS-1-dependent, cooperative, and TTSS-1-independent *S*.Tm invasion mechanisms in human epithelial, monocyte, and macrophage cells. The results reveal that *S*.Tm invasion of monocytes, similar to epithelial cells, comprises a mix of TTSS-1-dependent and cooperative invasion mechanisms, with TTSS-1-independent entry accounting for less than 1% of all events. Surprisingly, also invasion of macrophages is predominantly a TTSS-1-dependent process during the first minutes of bacterium-host cell interactions, while cooperative invasion is notably absent in this cell type. Finally, we identify a generic dependence on the effectors SopB, SopE, and SopE2 for TTSS-1-dependent *S*.Tm invasion of both monocytes and macrophages.

## RESULTS

### A framework for analyzing *S.*Tm host cell invasion mechanisms by barcoded consortium infections.

Barcoding of the bacterial chromosome enables detection of multiple strains in mixed consortium samples. Here, we took advantage of a set of seven unique 40-nucleotide tags (hereafter tagA to tagG [tagA-G]; see Fig. S1 in the supplemental material), each individually inserted into the *malXY* locus of isogenic *S*.Tm strains (36). The tag sequences have been used to evaluate bacterial population dynamics in animal models and shown not to confer any fitness defects (13, 36, 37).

**FIG 1:**
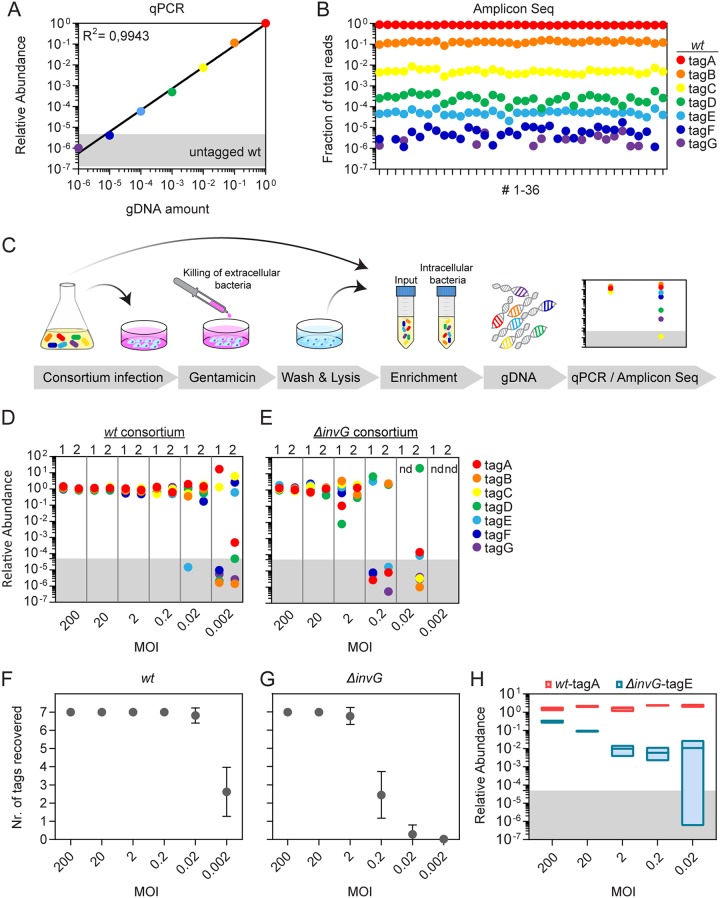
Barcoded consortium infections for host cell invasion studies. (A and B) Linear quantification of *S*.Tm chromosomal tags by qPCR and Amplicon Seq. (A) Standard curve for qPCR detection of tags in a mixed gDNA template comprising seven tagged *S*.Tm*^wt^* gDNA preparations in a 10-fold dilution series (tagA, 9 ng [10^0^]; tagB, 0.9 ng [10^−1^]; tagC, 0.09 ng [10^−2^]; tagD, 0.009 ng [10^−3^]; tagE, 0.0009 ng [10^−4^]; tagF, 0.00009 ng [10^−5^]; tagG, 0.000009 ng [10^−6^]). Gray shading indicates the range of unspecific signals when untagged gDNA was used as the qPCR template. (B) Amplicon Seq quantification of the seven tags in the same mixed gDNA template as in Fig. 1A, run in 36 parallel reactions with unique metabarcoded primer pairs (see Table S1 in the supplemental material). (C) Schematic representation of the protocol used for barcoded consortium infections of cultured host cells. (D and E) Assessment of stochastic tagged strain(s) loss across MOIs. A total of 150,000 HeLa cells were infected with a mixed barcoded consortium comprising either seven *S*.Tm*^wt^* (tagA-G) strains (D) or seven *S*.Tm*^ΔinvG^* (tagA-G) strains (E) for 20 min at the indicated MOIs. Graphs depict relative tag abundances in the intracellular *S*.Tm population by qPCR. Two replicates (replicates 1 and 2) are shown side by side. Gray shading indicates the detection limit. nd, no detectable bacteria. (F and G) *In silico* simulations of experiments in panels D and E (see Materials and Methods for details). The simulations were iterated 1,000 times for both the *S*.Tm*^wt^* (F) and the *S*.Tm*^ΔinvG^* (G) consortium infection. Results are shown as means ± SDs. (H) Assessment of the impact of cooperative *S*.Tm invasion. The graph depicts the relative tag abundances in the intracellular *S*.Tm population after HeLa cell coinfection (20 min, MOIs as indicated) with a mixed barcoded consortium comprising a 1:1 mix of *S*.Tm*^wt^*-tagA and *S*.Tm*^ΔinvG^*-tagE strains. Relative abundances were normalized to the inoculum. Results shown are means ± min/max values from three experiments. Gray shading indicates the detection limit.

10.1128/mBio.00603-19.1 FIG S1 Validation of chromosomal tag detection by qPCR and Amplicon Seq (supporting data for Fig. 1). (A) Schematic representation of the tag-containing *S.*Tm chromosomal locus (based on Grant et al. [36]). Primer binding sites for tag quantification by qPCR and Amplicon Seq are indicated. (B and C) Specificity of tag detection by qPCR. (B) gDNA from individual tagged S.Tm*^wt^* strains and from a mixed consortium comprising seven S.Tm*^wt^* strains (tagA-G) were amplified with specific primer pairs for each tag in parallel reactions. A heat-map based on Ct values is shown. (C) Five different *S*.Tm*^wt^* tagA-G barcoded consortia were generated with a gradual underrepresentation of the *S*.Tm*^wt^*-tagA strain (undiluted or diluted 10-, 25-, 50-, or 100-fold). gDNA from each consortium was extracted, and tagA-G abundances were quantified by qPCR. A heat-map based on Ct values is shown. Note that dilution of tagA is readily observable, whereas the Ct values for the other six strains remain constant. (D) High-confidence matching of Amplicon Seq results to individual chromosomal tags. The graph depicts the read distribution in the 36 replicate samples of the sequencing library generated for the Fig. 1B experiment. Total reads (dark gray) and reads that could be confidently assigned to a specific tag (light gray) are shown for each sample. Note that ≥95% of the total reads could be assigned to specific chromosomal tags. Download FIG S1, PDF file, 0.6 MB. Copyright © 2019 Di Martino et al. 2019 Di Martino et al. This content is distributed under the terms of the Creative Commons Attribution 4.0 International license.

As a basis for host cell invasion studies, we optimized two methods for quantification of tag abundance, using either quantitative qPCR for quick bench-side analysis or Amplicon Seq for larger sample sets (see Tables S1 and S2 and Fig. S1A in the supplemental material). Both methods were extensively validated for specificity and efficiency of detection (Fig. S1 and S2). Accurate quantification of strain abundances in consortia requires direct comparisons between tagA-G. Therefore, we generated a mixed genomic DNA (gDNA) template, comprising seven tagA-tagG gDNA preparations in a 10-fold dilution series. qPCR quantification of tags in the mixed sample resulted in a linear standard curve that agreed closely with the experimental dilutions (Fig. 1A). Amplification of unspecific background varied somewhat between primer pairs (Fig. S2), corresponding to relative abundance values of 1.5 × 10^−7^ to 5 × 10^−6^ (Fig. 1A, gray shading). When the same mixed gDNA template was analyzed by Amplicon Seq in 36 replicate reactions on a 20 million read chip, the results again agreed with the experimental dilutions (Fig. 1B). As expected, we did not detect tagG (10^−6^ dilution) reads in 21/36 replicates, and tagF (10^−5^) was also lost in 2/36 cases. All considered, we can by qPCR or Amplicon Seq stringently quantify tag abundances in a consortium, down to a conservative detection limit of 5 × 10^−5^.

10.1128/mBio.00603-19.2 FIG S2 Linear detection of all seven chromosomal tags by qPCR (supporting data for Fig. 1). (A to G) Individual standard curves for qPCR detection of each tag (tag A to G), using specific primer pairs (Table S1). Nine nanograms of gDNA from pure monocultures of each strain of seven strains *S*.Tm*^wt^* (tagA-G) were diluted in 10-fold serial dilutions (up to 10^−6^) and used to generate standard curves for each primer pair. Dotted lines indicate the unspecific signal for each primer pair when 9 ng of untagged gDNA was used as the template. R^2^ values are indicated in the bottom right corner of each panel. Download FIG S2, PDF file, 0.7 MB. Copyright © 2019 Di Martino et al. 2019 Di Martino et al. This content is distributed under the terms of the Creative Commons Attribution 4.0 International license.

10.1128/mBio.00603-19.9 TABLE S1 Chromosomal tag sequences and primers for qPCR and Amplicon Seq used in this study. Download Table S1, PDF file, 0.2 MB. Copyright © 2019 Di Martino et al. 2019 Di Martino et al. This content is distributed under the terms of the Creative Commons Attribution 4.0 International license.

10.1128/mBio.00603-19.10 TABLE S2 Bacterial strains used in this study. Download Table S2, PDF file, 0.4 MB. Copyright © 2019 Di Martino et al. 2019 Di Martino et al. This content is distributed under the terms of the Creative Commons Attribution 4.0 International license.

For host cell invasion studies, we developed an internally controlled gentamicin protection assay, using mixed consortia of tagged *S.*Tm strains as inoculum (Fig. 1C and Fig. S3A). To prepare samples for qPCR or Amplicon Seq analysis, the intracellular bacterial population can be extracted and enriched in a nonselective broth. A starting population that is too small may, however, result in stochastic loss of strains in the enriched culture. To validate the infection settings and assess the influence of multiplicity of infection (MOI) on stochastic loss, we infected HeLa cells (150,000 cells infected for 20 min) with a 1:1:1:1:1:1:1 pool of seven tagged *S*.Tm*^wt^* or seven tagged *S*.Tm*^ΔinvG^* strains at a range of MOIs (0.002 to 200). *S*.Tm*^ΔinvG^* lacks the outer membrane ring InvG protein, resulting in a nonfunctional TTSS-1 and a 500- to 1,000-fold-lower ability to invade HeLa cells in parallel comparisons (Fig. S3B). For each MOI, we assessed the total size of the intracellular bacterial population by plating and the distribution of tagged strains by qPCR. Detectable numbers (≥10 CFU) of *S*.Tm*^wt^* could be recovered down to an MOI of 0.002, whereas for the *S*.Tm*^ΔinvG^*-tagged consortium, plating failed to detect intracellular bacteria at an MOI of 0.2 and below (Fig. S3C and D). In keeping with these results, the *S*.Tm*^wt^* consortium exhibited loss of several tags at an MOI of 0.002 (Fig. 1D), while for the *S*.Tm^Δ*invG*^ consortium, tag loss occurred also at MOIs of 0.02 to 0.2 (Fig. 1E). Importantly, different sets of tags were lost in replicate experiments (Fig. 1D and E), highlighting stochastic strain loss at low MOIs. *In silico* simulations of the experiments in Fig. 1D and E (1,000 iterations) substantiated a reproducible loss of tags at MOIs of ≤0.002 for the *S*.Tm*^wt^* consortium and at an MOI of ≤0.2 for the *S*.Tm*^ΔinvG^* consortium (Fig. 1F and G). Hence, to avoid the risk of stochastic strain loss, MOIs lower than 0.2 should not be used under the present conditions. However, by scaling up the experimental size, tag loss at low MOIs could be further mitigated (Fig. S3E).

10.1128/mBio.00603-19.3 FIG S3 Experimental parameters for barcoded consortium infections of cultured host cells (supporting data for Fig. 1). (A) No observable fitness cost of chromosomal barcodes in rich LB. The pie charts depict the composition of barcoded *S*.Tm consortia used as inocula for experiments in Fig. 1D. A mixed consortium comprising seven S.Tm*^wt^* strains (tagA-G) was diluted and enriched overnight, and strain tag abundance in extracted gDNA was analyzed by qPCR. The relative abundance of each strain is plotted as a fraction of the total consortium. Shown are data for five independently generated consortia and the mean of all five data sets. Note that no strain is consistently over- or underrepresented. (B) Quantitative impact of *invG* deletion on *S*.Tm invasiveness. The graph shows enumeration of the total intracellular CFUs in HeLa cells infected with *S*.Tm*^wt^* or *S*.Tm*^ΔinvG^* for 20 min at the indicated MOIs. Results are shown as means ± SDs and are representative of four independent experiments. (C and D) Total intracellular *S*.Tm population sizes in HeLa cell infections shown in Fig. 1D and E. The graphs show enumeration of the total intracellular CFUs upon infection with the *S*.Tm*^wt^* (C) or *S*.Tm*^ΔinvG^* (D) consortium. Results are shown as means ± SDs and are representative of two independent experiments. (E) Influence of experimental scale on the risk of stochastic strain loss. HeLa cells were infected with a mixed barcoded consortium comprising three *S*.Tm*^wt^* (tagA, tagB, and tagC) strains and 4 *S*.Tm*^ΔinvG^* (tagD, tagE, tagF, and tagG) strains. Infections were performed using different numbers of host cells; i.e., in 24-well plates (75,000 cells/well), 12-well plate (150,000 cells/well), 6-well plate (360,000 cells/well), or T-25 flask (1,000,000 cells/flask), for 20 min at the indicated MOIs. The graph shows the relative tag abundances in the intracellular *S*.Tm population as analyzed by qPCR. Relative abundances were normalized to the inoculum. Note that stochastic loss of one or several *S*.Tm*^ΔinvG^* strains (gray shading indicates detection limit) becomes less severe as the total number of host cells increases. (F and G) Microscopy-based assessment of cooperative *S*.Tm invasion. (F) Representative images of HeLa cells coinfected with a 1:1 mix of *S*.Tm*^wt^* (helper [H]):*S*.Tm*^ΔinvG^*-*ssaG*GFP (reporter [R]), or *S*.Tm*^ΔinvG^* (H):*S*.Tm*^ΔinvG^*-*ssaG*GFP (R) for 20 min at an MOI of 200. Scale bars, 50 μm. (G) Microscopy-based enumeration of intracellular *S*.Tm*^ΔinvG^*-*ssaG*GFP reporter foci at the indicated total MOIs. Results are shown as means ± SDs and are representative of two experiments. (H) Assessment of cooperative *S*.Tm invasion by plating of gentamicin-protected bacteria. The graph shows enumeration of Km^r^ intracellular CFUs in HeLa cells coinfected with a 1:1 mix of either *S*.Tm*^wt^* (H):*S*.Tm*^ΔinvG^*-Km^R^ (R) or *S*.Tm*^ΔinvG^* (H):*S*.Tm*^ΔinvG^*-Km^R^ (R) for 20 min at the indicated total MOIs. Results are shown as means ± SDs and are representative of three experiments. Download FIG S3, PDF file, 0.4 MB. Copyright © 2019 Di Martino et al. 2019 Di Martino et al. This content is distributed under the terms of the Creative Commons Attribution 4.0 International license.

*S.*Tm enters cultured epithelial cells predominantly by TTSS-1 effector induction of actin-rich ruffles (2). The ruffles also promote macropinocytotic uptake of proximal bystander bacteria, a phenomenon denoted cooperative invasion (24, 25, 38). Since the chance of another bacterium arriving at a ruffle increases with bacterial density, the frequency of cooperative invasion is predicted to increase with MOI. In barcoded consortium infections, cooperative invasion could influence the experimental outcome and lead to overestimation of the invasion capacity of attenuated mutants. We addressed the impact of cooperative invasion by performing 1:1 dual strain coinfections in HeLa cells over a range of five total MOIs (0.02 to 200). At each MOI, we assessed the ability of an *S*.Tm*^wt^* helper strain to promote the entry of *S*.Tm*^ΔinvG^* (lacks TTSS-1 function). Both a microscopy-based assay and plating on selective agar showed that the presence of a coinfecting *S*.Tm*^wt^* strain dramatically potentiated the number of intracellular *S*.Tm*^ΔinvG^* at high MOIs (∼50- to 100-fold at an MOI of 200 and ∼10-20-fold at an MOI of 20 (Fig. S3F to H). In contrast, this cooperative effect was neglectable at low MOIs (i.e., 0.2 to 2; Fig. S3H). We next performed an analogous experiment, using a simple barcoded consortium comprising two strains, *S*.Tm*^wt^*-tagA and *S*.Tm*^ΔinvG^*-tagE. Again, *S*.Tm*^ΔinvG^*-tagE faired markedly better at high MOIs than at low MOIs (Fig. 1H). At an MOI of 200, *S*.Tm*^ΔinvG^*-tagE exhibited only ∼5-fold-lower intracellular abundance than *S*.Tm*^wt^*-tagA. By sharp contrast, at MOIs of 0.2 to 2, the *S*.Tm*^ΔinvG^*-tagE strain was ∼150- to 400-fold less abundant than *S*.Tm*^wt^*-tagA (Fig. 1H). This estimate agrees well with the actual invasion defect of the *S*.Tm*^ΔinvG^* strain in parallel single-strain infections (Fig. S3B). Consequently, cooperative invasion appears minimal at low MOIs (∼0.2 to 2) and gradually increases with inoculum size.

Taken together, these data (i) validate the use of barcoded consortium infections for *S*.Tm host cell invasion studies, (ii) delineate the boundary conditions for analysis by qPCR and Amplicon Seq, and (iii) provide a means to assess cooperative invasion by comparisons across a range of MOIs.

### Contribution of TTSS-1-dependent, cooperative, and TTSS-1-independent *S*.Tm entry mechanisms in diverse host cell types.

In the course of its *in vivo* infection cycle, *S*.Tm can enter a wide variety of host cell types, including absorptive gut epithelial cells, M cells, goblet cells, monocytes, macrophages, dendritic cells, neutrophils, and lymphocytes (5, 9–12). Invasion of epithelial cells is viewed as a TTSS-1-driven active process, whereas entry into macrophages is presumed to occur largely by passive (TTSS-1-independent) uptake. However, it remains unclear whether *S*.Tm invasion mechanisms with unique features drive the entry process in each distinct host cell type, and furthermore to what extent cooccurring (i) TTSS-1-dependent (hereafter “TTSS-1”), (ii) cooperative, and (iii) TTSS-1-independent (hereafter “non-TTSS-1”) invasion mechanisms contribute in each case. The internally controlled conditions achieved by barcoded consortium infections provide an unprecedented opportunity to resolve such questions.

**FIG 2:**
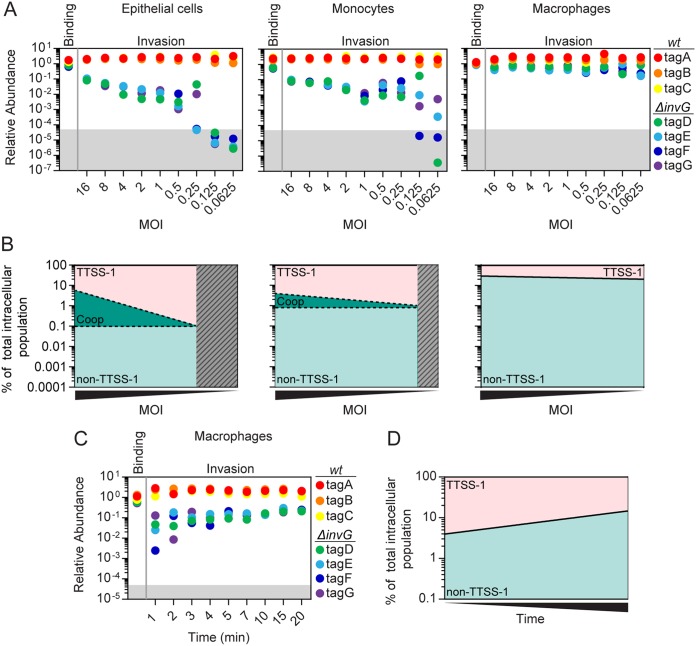
Quantification of *S*.Tm host cell binding and invasion mechanisms across cell types. HeLa epithelial cells, U937 monocytes, and U937-derived macrophages were infected with a mixed barcoded consortium comprising three *S*.Tm*^wt^* (tagA, tagB, and tagC) and four *S*.Tm*^ΔinvG^* (tagD, tagE, tagF, and tagG) strains at the indicated MOIs for 20 min (A and B) or 1 to 20 min (C and D). For binding assays, cells were pretreated with cytochalasin D prior to infection, and the gentamicin step was omitted prior to enrichment (see Fig. 1C). (A) Quantification of relative tag abundances in the intracellular *S*.Tm population in HeLa epithelial cells (left panel), U937 monocytes (middle panel), and U937-derived macrophages (right panel) as analyzed by qPCR. Relative abundances were normalized to the inoculum. (B) Area plots showing a regression (nonlinear fit) analysis of data in panel A, based on the mean values of all replicates for *S*.Tm*^wt^* (TTSS-1 positive) and *S*.Tm*^ΔinvG^* (TTSS-1 negative) strains (see Materials and Methods for details). Gray hatching indicates MOIs where stochastic loss of strains was observed. Three mechanisms of invasion could be resolved and estimated: TTSS-1 (pink), cooperative (Coop; dashed lines, green area), and non-TTSS-1 (turquoise) entry. (C and D) Pronounced TTSS-1 invasion of macrophages in the first minutes of host cell encounter. U937-derived macrophages were infected at an MOI of 1 over the indicated range of time points. (C) Quantification of relative tag abundances in the intracellular *S*.Tm population as analyzed by qPCR. Relative abundances were normalized to the inoculum. (D) Area plot showing a regression analysis (nonlinear fit) of data from panel C.

Towards this aim, we employed a 1:1:1:1:1:1:1 barcoded consortium comprising three *S*.Tm*^wt^* (tagA, tagB, and tagC) and four *S*.Tm*^ΔinvG^* (tagD, tagE, tagF, and tagG) strains to assess invasion mechanisms in three typical human cell models—epithelial (HeLa), monocyte (U937), and macrophage (PMA-differentiated U937) cells. Each cell type was infected for 20 min over a tightly spaced range of MOIs (16 – 8 – 4 – 2 – 1 – 0.5 – 0.25 – 0.125 – 0.0625). We reasoned that this setup would permit stringent quantification of the frequency of TTSS-1, cooperative, and non-TTSS-1 invasion events in each context. First, the consortium contained three or four replicates of each genotype. From this, a robust intracellular abundance value could be derived for TTSS-1-invading (*S*.Tm*^wt^*) and non-TTSS-1-invading (*S*.Tm*^ΔinvG^*) bacteria under internally controlled conditions. Second, the replicates also allowed surveillance of stochastic loss of strain(s) (evident from random loss of one or more tags) that could bias the results. Last, by comparing the mean relative abundance of *S*.Tm*^ΔinvG^* in the intracellular bacterial population between high and low MOIs, we could estimate cooperative invasion for each cell type. All samples were analyzed by both qPCR and Amplicon Seq, and the two methods were found to yield essentially identical results (Fig. 2A and Fig. S4A).

**FIG 3:**
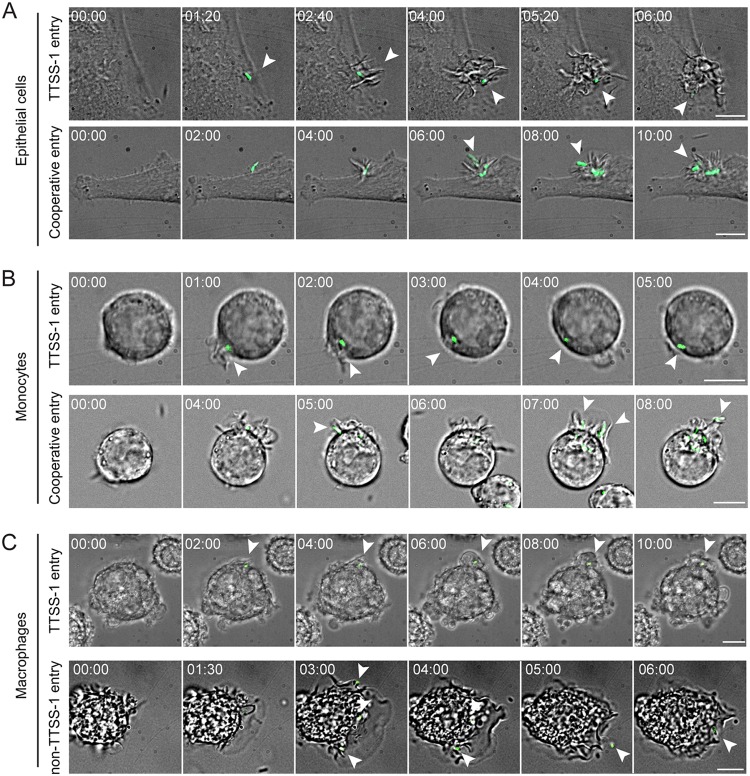
Examples of cooccurring *S*.Tm invasion mechanisms by live microscopy. HeLa epithelial cells, U937 monocytes, and U937-derived macrophages were infected with an *S*.Tm*^wt^*-GFP strain at MOIs of 10 to 50. Infections were imaged for 20 to 40 min, and images were taken every 20 to 30 s, using a confocal spinning disc microscope. DIC and GFP channel overlays are shown. (A) Representative montages of HeLa cell infections, exemplifying *S*.Tm invasion events consistent with TTSS-1 entry or cooperative entry. (B) Representative montages of U937 monocyte infections, showing *S*.Tm invasion events consistent with TTSS-1 entry or cooperative entry. (C) Representative montages of U937-derived macrophage infections, showing *S*.Tm invasion events consistent with TTSS-1 entry or non-TTSS-1 entry. Time is given in the minute:second format. Bars, 10 μm.

10.1128/mBio.00603-19.4 FIG S4 TTSS-1, cooperative, and non-TTSS-1 *S*.Tm invasion mechanisms across cell types (supporting data for Fig. 2 and 3). (A) HeLa epithelial cells, U937 monocytes, and U937-derived macrophages were infected with a mixed barcoded consortium comprising three *S*.Tm*^wt^* (tagA, tagB, and tagC) strains and four *S*.Tm*^ΔinvG^* (tagD, tagE, tagF, and tagG) strains as described in the legend to Fig. 2A. The graphs show quantification of the relative tag abundances in the binding and intracellular *S*.Tm populations in HeLa cells (left panel), U937 monocytes (middle panel), and U937-derived macrophages (right panel), as analyzed by Amplicon Seq. Relative abundances were normalized to the inoculum. Note that these results fully support the parallel analysis of the same samples done by qPCR (Fig. 2A). (B) Total intracellular *S*.Tm population sizes in U937-derived macrophages infected with *S*.Tm*^wt^* or *S*.Tm*^ΔinvG^* for 20 min at the indicated MOIs. Results are shown as means ± SDs and are representative of two independent experiments. (C) Assessment of host cell lysis during early *S*.Tm invasion. HeLa epithelial cells, U937 monocytes, and U937-derived macrophages were infected with *S*.Tm*^wt^* at the indicated MOIs. Propidium iodide uptake was analyzed by live microscopy at 1 h p.i. Results are shown as means ± SDs. The assay was validated by the addition of 0.15% saponin to the three cell types, which in all cases resulted in close to 100% PI-positive (PI+) cells. Download FIG S4, PDF file, 0.6 MB. Copyright © 2019 Di Martino et al. 2019 Di Martino et al. This content is distributed under the terms of the Creative Commons Attribution 4.0 International license.

*S*.Tm host cell invasion is preceded by a cell surface binding step that involves bacterial adhesins and the TTSS-1 apparatus. In line with previous studies (39), ablation of TTSS-1 function (*S*.Tm*^ΔinvG^*) resulted in only a marginal (≤2-fold) decrease in host cell binding for all three cell types (Fig. 2A and Fig. S4A). In the intracellular population, the relative abundance of *S*.Tm*^ΔinvG^* strains varied greatly, however, depending both on the MOI and the host cell type (Fig. 2A and Fig. S4A).

As expected, the invasion efficiency of *S*.Tm*^ΔinvG^* showed a clear correlation with MOI in epithelial (HeLa) cells. At an MOI of 16, *S*.Tm*^ΔinvG^* strains were on average 20-fold less abundant than *S*.Tm*^wt^*, whereas at lower MOIs (0.5 to 1), this difference was ∼200- to 400-fold. At an MOI of 0.25 and lower, stochastic loss of some *S*.Tm*^ΔinvG^* replicates was noted (Fig. 2A, left panel). Surprisingly, a highly similar pattern was also noted for *S*.Tm invasion of U937 monocytes (Fig. 2A, middle panel). By sharp contrast, *S*.Tm*^ΔinvG^* was only ∼3-fold less abundant than *S*.Tm*^wt^* across all MOIs for invasion of differentiated U937 macrophages (Fig. 2A, right panel). In addition, no stochastic loss of strains was noted here at lower inoculum densities, which can be explained by a larger total intracellular population of *S*.Tm*^ΔinvG^* upon macrophage infection (Fig. 2A, right panel, and Fig. S4B).

To conceptualize the data, we calculated the relative intracellular proportions of *S*.Tm*^wt^* and *S*.Tm*^ΔinvG^* bacteria for each condition, using all replicate values from the qPCR analysis. Samples exhibiting stochastic loss of strains were omitted (hatched areas in Fig. 2B), and a simple regression analysis was performed on the remaining data (Fig. 2B). These estimates highlight that a TTSS-1 *S*.Tm invasion mechanism(s) dominates entry into epithelial cells (93 to 99.99% of all events; Fig. 2B, left panel) and in monocytes (96 to 99.5%; Fig. 2B, middle panel). Non-TTSS-1 uptake contributed with ∼0.1% and ∼0.8%, respectively (Fig. 2B). Moreover, cooperative entry occurred in both cell types and accounted for ≥7% of all invasion events in epithelial cells and ≥4% in monocytes at the highest MOI (Fig. 2B). It should be noted that these values represent minimal estimates of cooperative invasion (dashed bounds), since they rely on the assumptions that (i) cooperative invasion is completely absent at the lowest possible MOI (as validated for HeLa cells in Fig. S3H) and that (ii) cooperative helping effects between *S*.Tm*^wt^* strains have a modest quantitative impact. Nevertheless, our data resolve and quantify three cooccurring *S*.Tm invasion mechanisms in epithelial cells and monocytes.

The results further show that entry into macrophages occurs by a mix of TTSS-1 and non-TTSS-1 mechanisms (∼75% TTSS-1 and ∼25% non-TTSS-1 events across MOIs; Fig. 2B, right panel). These data agree with the higher phagocytic potential of macrophages. However, when infections were conducted at a constant MOI (MOI of 1) but over a range of shorter infection times (1 to 20 min), up to ∼96% of all invasion events were of the TTSS-1 sort (Fig. 2C and D). Hence, although macrophages have a high phagocytic capacity, *S*.Tm entry is predominantly a bacterium-driven process during the first few minutes of interaction. Of further note, no contribution could be assigned to cooperative invasion in this cell type (Fig. 2B to D).

*S*.Tm entry into phagocytes may over time result in cell death in a TTSS-1-dependent manner (30). To visualize the cooccurring invasion mechanisms and survey for possible effects on the host cells, we employed live microscopy of the three cell types infected with GFP-expressing *S*.Tm*^wt^* (Table S2). In both HeLa epithelial cells and U937 monocytes, we could frequently observe typical TTSS-1 invasion events, morphologically defined by bacterial binding to a previously unperturbed cell followed by ruffle induction (Fig. 3A and B; see also Movies S1A and S2A in the supplemental material). Cooperative invasion events, i.e., the capture of secondary bacteria in membrane ruffles triggered by a primary invader, could also be visualized at rarer frequency (Fig. 3A and B and Movies S1B and S2B). We could not with confidence detect non-TTSS-1 entry (i.e., entry in the absence of *S*.Tm-induced ruffles) into either of these cell types, which is in line with the low quantitative estimates (Fig. 2A and B and Fig. 3A and B). In contrast, *S*.Tm entry events in U937 macrophages partitioned into a frequent TTSS-1 type and a slightly less frequent non-TTSS-1 type, i.e., where the dynamic macrophage membrane engulfed *S*.Tm without signs of bacterium-triggered perturbations (Fig. 3C and Movie S3A and B). In line with previous studies (30), some monocytes and macrophages begun displaying early signs of cell death (e.g., circumferential blebbing), but cell lysis did not become pronounced within the short time span of the experiments (Fig. S4C). In conclusion, live imaging can exemplify the cooccurring *S*.Tm invasion mechanisms quantified by barcoded infections in each host cell type.

10.1128/mBio.00603-19.6 MOVIE S1 Live imaging series of *S*.Tm*^wt^*-GFP-infected HeLa epithelial cells, showing invasion events consistent with TTSS-1 entry (A) or cooperative entry (B) (supporting data for Fig. 3). The movies comprise an overlay of DIC and GFP channels. Time is given in the minute:second format. Scale bar, 10 μm. Download Movie S1, AVI file, 1.5 MB. Copyright © 2019 Di Martino et al. 2019 Di Martino et al. This content is distributed under the terms of the Creative Commons Attribution 4.0 International license.

10.1128/mBio.00603-19.7 MOVIE S2 Live imaging series of *S*.Tm*^wt^*-GFP-infected U937 monocytes, showing invasion events consistent with TTSS-1 entry (A) or cooperative entry (B) (supporting data for Fig. 3). The movies comprise an overlay of DIC and GFP channels. Time is given in the minute:second format. Scale bar, 10 μm. Download Movie S2, AVI file, 1.1 MB. Copyright © 2019 Di Martino et al. 2019 Di Martino et al. This content is distributed under the terms of the Creative Commons Attribution 4.0 International license.

10.1128/mBio.00603-19.8 MOVIE S3 Live imaging series of *S*.Tm*^wt^*-GFP-infected U937-derived macrophages, showing invasion events consistent with TTSS-1 entry (A) or non-TTSS-1 entry (B) (supporting data for Fig. 3). The movies comprise an overlay of DIC and GFP channels. Time is given in the minute:second format. Scale bar, 10 μm. Download Movie S3, AVI file, 2.3 MB. Copyright © 2019 Di Martino et al. 2019 Di Martino et al. This content is distributed under the terms of the Creative Commons Attribution 4.0 International license.

### The combined actions of SopB, SopE, and SopE2 drive TTSS-1 invasion of cultured epithelial, monocyte, and macrophage cells.

TTSS-1-dependent invasion has been extensively studied in HeLa cells. Four of the TTSS-1 effectors appear to mainly fuel the invasion process. The phosphatidyl inositol phosphatase SopB and the highly homologous Rho GEFs SopE and SopE2 drive Arp2/3-dependent actin nucleation, resulting in membrane ruffling, while SipA collaborates with the translocon component SipC to stabilize filamentous actin around the invading bacterium (2). Additional TTSS-1 effectors (e.g., AvrA, SptP) (22, 23, 40) have also been characterized, but they do not seem to significantly impact HeLa cell invasion. It remains unclear whether unique or similar effector sets drive TTSS-1-dependent invasion across host cell types.

**FIG 4:**
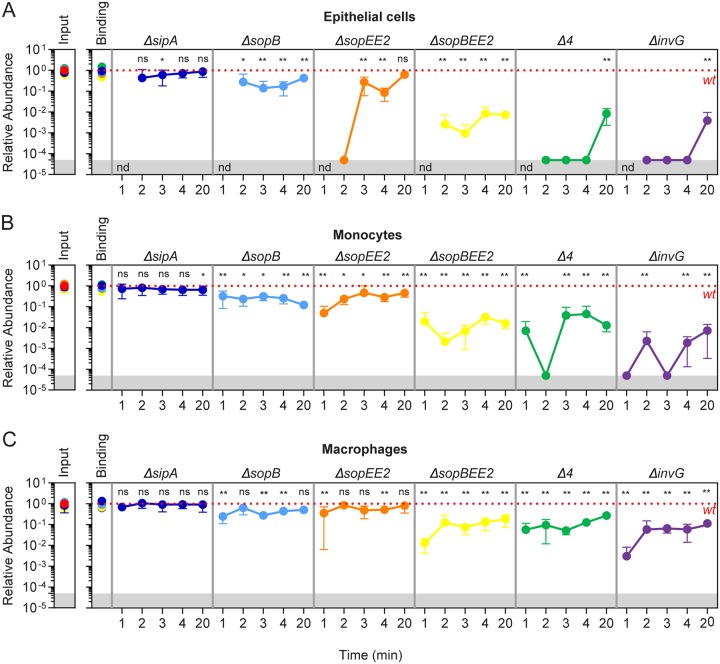
Contribution of TTSS-1 effectors to *S*.Tm invasion of distinct host cell types. HeLa epithelial cells, U937 monocytes, and U937-derived macrophages were infected with a mixed barcoded consortium comprising *S*.Tm*^wt^*-tagA, *S*.Tm*^ΔsipA^*-tagF, *S*.Tm*^ΔsopB^*-tagE, *S*.Tm*^ΔsopEE2^*-tagB, *S*.Tm*^ΔsopBEE2^*-tagC, *S*.Tm*^Δ4^*-tagD, and *S*.Tm*^ΔinvG^*-tagG strains at an MOI of 1 for 1, 2, 3, 4, or 20 min. For binding assays, cells were pretreated with cytochalasin D prior to infection, and the gentamicin step was omitted prior to enrichment. (A to C) Quantification of relative tag abundances in input, the host cell binding population, or the intracellular *S*.Tm population in HeLa epithelial cells (A), U937 monocytes (B), and U937-derived macrophages (C) as analyzed by qPCR. Relative abundances in binding and intracellular bacterial populations were normalized to the inoculum (input) and subsequently to the *S*.Tm*^wt^* reference strain (*S*.Tm*^wt^*-tagA; indicated by the dotted red line). For the intracellular population data, the behavior of each mutant strain is illustrated in a separate panel. Results are shown as mean ± SD from three experiments. Values that are significantly different from the value for the *S*.Tm*^wt^*-tagA control strain by one-way ANOVA with Tukey’s multiple-comparison test are indicated by asterisks as follows: *, *P* < 0.05, **, *P* < 0.01.

Here, we used barcoded consortium infections to quantify the contribution of effectors to TTSS-1 invasion in epithelial, monocyte, and macrophage cells. For the inoculum, we generated a 1:1:1:1:1:1:1 mix of the seven strains *S*.Tm*^wt^*, *S*.Tm*^ΔsipA^*, *S*.Tm*^ΔsopB^*, *S*.Tm*^ΔsopEE2^*, *S*.Tm*^ΔsopBEE2^*, *S*.Tm*^Δ4^* (*ΔsopBEE2ΔsipA*), and *S*.Tm*^ΔinvG^*, each harboring one tag from tags A-G (Table S2). We confirmed that all strains were equally represented in the inoculum and that no strain exhibited a growth defect in the enrichment broth (Fig. S5A). The infection experiments were set up to maximize the percentage of TTSS-1 invasion events and minimize any contribution of cooperative entry. Consequently, we performed infections at an MOI of ∼1 and included multiple short time points (1, 2, 3, 4, and 20 min of infection). Samples were analyzed by both qPCR and Amplicon Seq, which yielded essentially identical results (Fig. 4A to C and Fig. S5B to D).

10.1128/mBio.00603-19.5 FIG S5 Impact of TTSS-1 effectors on *S*.Tm invasion of distinct host cell types (supporting data for Fig. 4). (A) No observable fitness cost in rich LB for either of the barcoded consortium strains carrying mutations in TTSS-1 effectors. The pie charts depict the composition of barcoded *S*.Tm consortia used as inocula for experiments in Fig. 4A to C or Fig. S5B to D. A mixed consortium comprising the seven strains *S*.Tm*^wt^-*tagA, *S*. Tm*^ΔsipA^*-tagF, *S*.Tm*^ΔsopB^*-tagE, *S*.Tm*^ΔsopEE2^*-tagB, *S*.Tm*^ΔsopBEE2^*-tagC, *S*.Tm*^Δ4^*-tagD, and *S*.Tm*^ΔinvG^*-tagG was diluted and enriched overnight, and strain tag abundance in extracted gDNA was analyzed by qPCR. The relative abundance of each strain is plotted as a fraction of the total consortium. Shown are data for nine independently generated consortia and the mean of all nine data sets. Note that no strain is consistently over- or underrepresented. (B and D) HeLa epithelial cells, U937 monocytes, and U937-derived macrophages were infected with the mixed barcoded consortium comprising *S*.Tm*^wt^*-tagA, *S*.Tm*^ΔsipA^*-tagF, *S*.Tm*^ΔsopB^*-tagE, *S*.Tm*^ΔsopEE2^*-tagB, *S*.Tm *^ΔsopBEE2^*-tagC, *S*.Tm*^Δ4^*-tagD, and *S*.Tm*^ΔinvG^*-tagG strains as described in the legend to Fig. 4A. The graphs show quantification of the relative tag abundances in the input, the host cell binding population, and the intracellular *S*.Tm population in HeLa epithelial cells (B), U937 monocytes (C), and U937-derived macrophages (D) as analyzed by Amplicon Seq. Relative abundances in the binding and intracellular bacterial populations were normalized to the inoculum (input) and subsequently to the *S*.Tm*^wt^* reference strain (*S*.Tm*^wt^*-tagA, indicated by dotted red line). For the intracellular population data, the behavior of each mutant strain is illustrated in a separate panel. Results are shown as means ± SDs from at least two experiments. Note that these results fully support the parallel analysis of the same samples done by qPCR (Fig. 4A to C). Download FIG S5, PDF file, 0.7 MB. Copyright © 2019 Di Martino et al. 2019 Di Martino et al. This content is distributed under the terms of the Creative Commons Attribution 4.0 International license.

All strains in the consortium were capable of binding HeLa cells with minor differences (Fig. 4A). Analysis of the intracellular population revealed the invasion capacity of each strain at a given time point. Here, *S*.Tm*^Δ4^* and *S*.Tm*^ΔinvG^* behaved virtually identically; none of the strains were recovered for the short-term (2- to 4-min) infections, and both displayed a similar ≥150-fold-lower intracellular abundance than *S*.Tm*^wt^* upon 20-min infections (Fig. 4A and Fig. S5B). This confirms that at most four effectors (SopB, SopE, SopE2 and SipA) explain TTSS-1-dependent invasion of HeLa cells. Internal comparisons between single and multiple effector mutants allowed us to further tease apart the contribution of each effector combination. Deletion of SipA (*S*.Tm*^ΔsipA^*) did not result in an appreciable attenuation of invasiveness (Fig. 4A and Fig. S5B). In contrast, deletion of SopB (*S*.Tm*^ΔsopB^*) or SopEE2 (*S*.Tm*^ΔsopEE2^*) resulted in a significant, but partial, attenuation, while the combined deletion of these three effectors (*S*.Tm*^ΔsopBEE2^*) caused >100-fold-lower invasion capacity. Moreover, *S*.Tm*^ΔsopBEE2^* was only modestly better than the *S*.Tm*^Δ4^* mutant (i.e., lacking also SipA) (Fig. 4A and Fig. S5B). Hence, TTSS-1 invasion of HeLa cells during the first 2 to 20 min relies on the combined action of SopB/SopE/SopE2 (>99%), with only a minimal contribution of SipA and no detectable impact of other effectors. These findings validate and extend previous work (33).

We next used the same consortium and setup to infect monocytes and macrophages. Here, intracellular bacteria were recovered already at 1 min postinfection (p.i.), which suggests that the invasion process can be completed even quicker than in HeLa cells (compare Fig. 4B and C to Fig. 4A). Moreover, as expected from the results in Fig. 2, non-TTSS-1 invasion (defined by the relative abundance of *S*.Tm*^ΔinvG^*) contributed more to the total intracellular *S*.Tm pool in macrophages than in HeLa cells and monocytes (Fig. 4A to C). Despite these differences, the pattern of effector dependence for TTSS-1 invasion was strikingly similar across the three host cell types (Fig. 4A to C and Fig. S5B to D). In both monocytes and macrophages, a neglectable contribution could be assigned to SipA, whereas deletion of SopB, SopEE2, or SopBEE2 resulted in progressive attenuation of invasiveness (Fig. 4B and C). *S*.Tm*^ΔsopBEE2^* showed essentially the same attenuation as *S*.Tm*^Δ4^*, further in line with a minimal contribution of SipA. Finally, comparisons between *S*.Tm*^Δ4^* and *S*.Tm*^ΔinvG^* revealed that effectors other than SopB, SopE, SopE2, or SipA (e.g., AvrA, SptP, or the translocon component SipC), may on their own at best have a minor impact during the first minutes of *S*.Tm invasion of monocytes/macrophages (Fig. 4B and C and Fig. S5C and D). In macrophages, such effectors are estimated to account for ∼5% of the invasion events in 1 min infection (Fig. 4C).

From these data, we conclude that TTSS-1 invasion of host cells as diverse as cultured epithelial cells, monocytes, and macrophages relies on a remarkably similar SopB/SopE/SopE2 effector combination.

## DISCUSSION

Consortia of genetically tagged, but otherwise identical, bacterial strains have emerged as a powerful tool to assess pathogen population dynamics in animal model infections with *Salmonella* (13, 36, 37, 41), as well as other microbes (42–45). In this work, we have adapted the use of bacterial barcoded consortia to studies of *S*.Tm host cell invasion and to competitive infections with mixes of wild-type and virulence gene mutant strains. Taken together, we show that barcoded consortium infections provide a flexible platform for mechanistic studies in cellular microbiology, with several advantages over classical techniques. These advantages include the following: (i) internally normalized infection conditions, (ii) stringent monitoring of experimental noise; (iii) possibility to compare the invasive behavior of many strains on the single-minute time scale; and (iv) the scalability of the method, particularly when combined with Amplicon Seq. Here, we have probed bacterial host cell binding and invasion mechanisms, but this approach is in fact much more versatile than that. Barcoded consortium infections will also permit the tracing of competing strains over the subsequent stages of host cell colonization, i.e., intracellular survival, trafficking, replication, and egress (46, 47).

Salmonella enterica serovars, including *S.*Tm, infect a number of warm-blooded host species and can invade diverse host cell types (5, 10, 48). Still, the bulk of mechanistic entry studies have been performed in a few epithelial cell lines. Hence, how *S*.Tm invasion mechanism(s) varies with infection context remains an incompletely explored topic. We found that the proportions of TTSS-1-dependent, cooperative, and TTSS-1-independent invasion events vary considerably between contexts, dictated both by the host cell type infected, the timing of the infection, and the MOI. In the experimental settings tested (and with the inocula cultivated under SPI-1-inducing conditions in the absence of opsonization), TTSS-1-dependent invasion accounted for ∼75 to ∼99.9% of all invasion events, cooperative invasion for ∼0 to ≥7%, and non-TTSS-1 invasion for ∼0.1 to ∼25%. Notably, *S*.Tm entry events in epithelial cells and monocytes showed a highly similar MOI-dependent distribution between the three invasion mechanisms. In contrast, these proportions did not depend on the MOI during infection of macrophages, and we failed to detect cooperative invasion for this cell type. Cooperative *S*.Tm invasion depends on the TTSS-1 triggering of large membrane ruffles, physical obstacles that increase the likelihood for motile bacteria to dock and enter the same membrane region (24). It appears plausible that the high degree of steady-state membrane ruffling in macrophages, in contrast to epithelial cells and immature monocytes, makes any cooperative effect of TTSS-1-induced ruffles neglectable in this cell type.

The conditions in the gut lumen cause *S*.Tm to express both flagellar and SPI-1 genes (49–51), which explains why bacteria reaching the intestinal epithelium as a rule are TTSS-1 primed. *S*.Tm that breach the epithelial barrier can retain expression of TTSS-1 and effectors in the lamina propria (51) or in systemic organs (52, 53), where the bacteria meet a wide range of immune cells. Moreover, gut luminal (and therefore TTSS-1-expressing) *S*.Tm may come in contact with transepithelial dendritic cell extensions (54) or encounter phagocytes that migrate into the inflamed gut at later infection stages (12, 13). The data presented here show that TTSS-1-dependent invasion can account for up to 96% of *S*.Tm entry events in macrophages during the first minutes of interaction. Consequently, it seems plausible that TTSS-1-triggered entry into professional phagocytes also has a significant impact *in vivo*. Finally, our data do not lend support to a broadly generalizable impact of “atypical” non-TTSS-1 entry mechanisms (27–29) across host cell types. Barcoded consortium infections will, however, provide a powerful means to search for specific host cells or conditions, where such mechanisms could dominate the entry process.

A large body of literature has uncovered the biochemical activities of the effectors that drive TTSS-1-dependent entry (2). However, the relative contribution of each effector during invasion across divergent epithelial and blood-derived cell types has not been systematically addressed. We have here begun such comparative studies. The data reveal that TTSS-1-dependent *S*.Tm invasion of human monocytes and macrophages rely on a highly similar effector program as the one used for ruffle-mediated entry in epithelial cell lines. Consequently, actin modulation through the combined action of the lipid phosphatase SopB (17–19) and the Rho-GEFs SopE (20) and SopE2 (21) constitutes a generic program for TTSS-1-dependent entry across epithelial and blood-derived cell types. It remains to be explored whether this will also hold true for other host cell types and for primary cells/tissues *in vivo*. Notably, recent work hints that *S*.Tm invasion of the mouse gut absorptive epithelium may depend on different TTSS-1 effectors than what has been observed for epithelial cell lines (55; Böck D., Fattinger S.A., Di Martino M.L., Deuring S., Furter M., Kreibich S., Bosia F., Müller A.A., Nguyen B.D., Rohde M., Pilhofer M., Hardt W.-D., Sellin M.E., submitted for publication). These observations warrant further studies of the impact of host cell context on *S*.Tm invasion mechanism(s) *in vivo*.

An emerging trend in infection biology is the replacement of simplistic immortalized cell line infection models with more physiological counterparts, such as organoids (56), multiple host cell type cocultures (57), tissue explants (58), and advanced whole-animal models (59). Whereas such models better recapitulate the host cell and tissue features encountered during a natural infection, they are inherently costly, are subject to large variability between replicate wells/tissues/animals, and allow limited scalability. This restricts the use of classical techniques for bacterium-host cell interaction studies (e.g., gentamicin protection assays combined with CFU plating), since they rely on parallel analysis of large numbers of single strain infections. We foresee that barcoded consortium infections, as delineated here, will provide an invaluable tool for cost-effective, stringent, and scalable analysis of bacterium-host cell interplay in such next-generation experimental models.

## MATERIALS AND METHODS

### Bacterial strains.

Bacterial strains are listed in Table S2 in the supplemental material. Construction of barcoded mutants was performed by P22 transduction. The tags were transferred from donor *S*.Tm strains into the relevant mutants, followed by selection on LB agar containing 12.5 μg/ml chloramphenicol. Construction of the *S.*Tm*^ΔinvG^-Km^R^* was performed by transferring the *ΔinvG* deletion from *S*.Tm 14028-*ΔinvG* (60) into an *S.*Tm*^wt^* strain, followed by selection on LB agar containing 50 μg/ml kanamycin. The *S.*Tm*^ΔsopB^*-tagE strain was constructed by transferring the *ΔsopB* deletion from *S*.Tm 14028-*ΔsopB* (60) into *S.*Tm*^wt^*-tagE by an identical approach. The *S.*Tm*^ΔsopEE2^*-tagB strain was constructed in two steps. First, tagB from *S*.Tm*^wt^*-tagB was transferred into an *S.*Tm*^ΔsopE^* strain, and subsequently the *ΔsopE2* deletion from *S*.Tm 14028-*ΔsopE2* (60) was transferred into the *S.*Tm*^ΔsopE^*-tagB intermediate, followed by selection on kanamycin. All strains were verified by PCR.

### Mammalian cell culture.

HeLa epithelial cells (ATCC CCL-2) were grown in DMEM GlutaMAX (catalog no. 31966-021; Gibco) supplemented with 10% heat-inactivated fetal bovine serum (FBS) at 37°C and 10% CO_2_. U937 monocytes (ATCC CRL-1593.2) were grown in RPMI 1640 GlutaMAX (catalog no. 72400054; Gibco) supplemented with 10% heat-inactivated fetal bovine serum (FBS) at 37°C and 5% CO_2_. Cultures were passaged two or three times each week. For routine passaging, 100 IU/ml penicillin and 100 μg/ml streptomycin were added to the medium, but antibiotics were omitted during infection experiments. For macrophage differentiation, U937 monocytes were seeded into 12-well plates and differentiated by adding 50 nM phorbol-12-myristate-13-acetate (PMA) (catalog no. P8139; Sigma) to the culture medium for 48 h. The medium was subsequently replaced with PMA-free medium and changed every 48 h.

### Bacterial infections.

The indicated numbers of HeLa cells were seeded in 12- or 6-well (or larger vessels for Fig. S3E) plates 24 h prior to infection. U937 monocytes were seeded (150,000 to 300,000/well) in precoated 12-well BAM (*b*iocompatible *a*nchor for cell *m*embranes) plates (61) 2 h prior to infection. For U937-derived macrophages, U937 monocytes were seeded (150,000/well) in 12-well plates, differentiated as described above, and infected on day 5 postdifferentiation. The indicated *S*.Tm strains were grown for 12 h in LB/0.3 M NaCl containing appropriate antibiotics, diluted 1:20, and subcultured for 4 h in 3 ml of the same medium without antibiotics. Inocula were cultured in a roller drum incubator. Strains were diluted in DMEM/10% FBS (for HeLa) or RPMI/10% FBS (for U937) to achieve the desired MOI. To generate barcoded consortia, the indicated tagged strains were mixed in equal ratios unless stated otherwise. After the addition of bacteria (without centrifugation), cultured cells were incubated at 37°C and 10% CO_2_ (HeLa) or 5% CO_2_ (U937) for 1 to 20 min as indicated. The culture medium was subsequently replaced with new medium containing 200 μg/ml gentamicin (catalog no. G1914; Sigma), and the cells were incubated for a total of 1 h (including infection time). At 1 h p.i., cells were washed with PBS and lysed in 0.1% sodium deoxycholate (catalog no. D6750; Sigma). For plating assays, the lysates were serially diluted and plated on LB agar containing appropriate antibiotics. For tag quantification, lysate bacterial populations were enriched overnight in 3 ml LB medium at 37°C. A diluted culture of the inoculum was also enriched and used as reference. A 1.5-ml portion of the bacterial culture was used to extract genomic DNA. For binding experiments, cells were treated with 1 μM cytochalasin D (catalog no. C8273; Sigma) 20 min prior to infection. Bacteria were added to the cells and incubated for 20 min. Cells were washed with PBS to remove unattached bacteria and processed as described above.

### Tag quantification by qPCR.

Genomic DNA from enriched cultures was extracted using the GenElute Bacterial Genomic DNA kit (catalog no. NA2110-1KT; Sigma). qPCR analysis with Maxima SYBR Green/ROX qPCR Master Mix (2×) (catalog no. K0222; Thermo Fisher Scientific) on a OneStepPlus instrument (Thermo Fisher Scientific) was performed using 9 ng of gDNA and tag-specific primers (Table S1 and Fig. S1A) based on reference 36. The relative abundance of each strain was normalized to the abundance in the inoculum. Standard curves were generated using gDNA from each tagged *S*.Tm*^wt^* strain (Fig. S2).

### Tag quantification by Amplicon Seq

Sequencing libraries were generated by amplifying each gDNA sample in a Phusion PCR (ThermoScientific) with primers in Table S1. The common forward primer harbors a sequencing adaptor and each reverse primer harbors a specific metabarcode, used for binning of the reads. Sequencing was performed on an Ion S5 XL System sequencer (Thermo Fisher Scientific) and the Ion 530 chip (20 million reads). Read counting was performed with an in-house-generated script. Briefly, the SamToFastq tool of the Java package Picard, version 2.18.10 (Broad Institute) was used to convert metabarcode-sorted IonTorrent.bam data files (containing reads), to the FASTAQ format. Reads were counted using fuzzy regular expressions allowing for four mismatches between the tag sequence and the read (the smallest Hamming distance between any tags was 15). The Python 3 script used to count the sequencing reads, *“*fastaq_read_counter.py,” is available from the GitHub repository at https://github.com/Oftatkofta/Barcoded_invasion.git.

### Generation of area plots for visualization of cooccurring invasion mechanisms.

At each MOI or time point, the respective mean abundance values for *S*.Tm*^wt^* (from the three tagged replicates) and *S*.Tm*^ΔinvG^* (from the four tagged replicates) strains were computed. These values were used to calculate the fraction of the intracellular *S*.Tm population that comprised TTSS-1-invading (*S*.Tm*^wt^*) and non-TTSS-1-invading (*S*.Tm*^ΔinvG^*) bacteria. Conditions where bottleneck effects were observed were excluded from the analysis (indicated by gray hatching). Cooperative invasion was estimated by a nonlinear fit regression of the mean abundance of *S*.Tm*^ΔinvG^* strains across MOIs. The plots are based on the assumption that cooperative invasion is neglectable at the lowest possible MOI (validated for HeLa cells in Fig. 1H and Fig. S3H). Furthermore, the plots contain a minimal estimate of cooperative invasion, since any cooperativity between *S*.Tm*^wt^* strains cannot be quantified.

### *In silico* simulation of host cell invasion.

The simulation was set up as a direct *in silico* adaptation of the barcoded invasion protocol (Fig. 1C). Briefly, the simulation created *n*_cells_ cell objects and randomly distributed *n*_bacteria_ bacterium objects among them. For each bacterium, there was a *P*_invade_ probability that the bacterium would invade a cell. Each bacterium had an associated tag, and each tag had its own *P*_invade_. For each iteration, a pool of bacterial objects was created with a tag composition that averaged to equal, but with an experimentally derived standard deviation. The simulation was run 1,000 times for each MOI. The output was the number of tags recovered (maximum seven). Coinvasion was assumed not to occur. The following parameters were used: *n*_cells_ = average 150,000, with an equal probability of a number between 125,000 and 175,000; *n*_bacteria_ = MOI × *n*_cells_, *P*_invade__wt = 1.146E−2, *P*_invade__*ΔinvG* = 1.018E−4 (experimentally derived from data in Fig. S3C and D). The standard deviation of the inoculum pool composition was 0.043 (experimentally derived from Fig. S3A). The Python 3 script used to run the simulation, *“*invasion_simulation.py,” is available from the GitHub repository at https://github.com/Oftatkofta/Barcoded_invasion.git.

### Fluorescence microscopy.

HeLa cells were seeded in eight-chamber slides (Labtek II [catalog no. 155409; Thermo Fisher Scientific]) and coinfected with an equal mix of *S*.Tm*^wt^* and *S*.Tm*^ΔinvG^*-*ssaG*GFP or *S*.Tm*^ΔinvG^* and *S*.Tm*^ΔinvG^*-*ssaG*GFP at different MOIs as indicated. At 4 h p.i., cells were fixed with 4% paraformaldehyde (PFA) for 15 min at 37°C, permeabilized with 0.5% Triton X-100 in PBS for 10 min, blocked in 10% normal goat serum (NGS) for 30 min, followed by DAPI (1:1,000) (catalog no. D9542; Sigma) and F-actin staining (1:200 phalloidin-Alexa Fluor 568 [catalog no. A12380; Molecular Probes]) for 40 min at room temperature. Fluorescence microscopy images were acquired on a Nikon Eclipse Ti microscope. For the analysis, infection foci in at least 1,200 cells were counted for each condition.

### Real-time confocal microscopy.

For live imaging, 10,000 cells were seeded in eight-chamber slides. Infections were performed at MOIs ranging from 10 to 50 with *S*.Tm^wt^-GFP. For analysis of host cell lysis, propidium iodide (1 μg/ml; Sigma) was added to the culture medium. Infections were imaged for 20 to 60 min as indicated (1 image/20 s for HeLa cells and monocytes; 1 image/30 s for macrophages) on a custom-built microscope, based on a Nikon Eclipse Ti2 core fitted with a 100×/1.45 NA oil objective, a X-Light-V2-LFOV spinning disk module (Crest), and a Prime 95B 25mm camera (Photometrics).
