# Supplementary figures and images for: Barcoded Consortium Infections Resolve Cell Type-Dependent Salmonella enterica Serovar Typhimurium Entry Mechanisms
Source: mBio. 2019 May 21;10(3):e00603-19. doi: 10.1128/mBio.00603-19 (PMC6529635; doi:10.1128/mBio.00603-19)

Figure S1

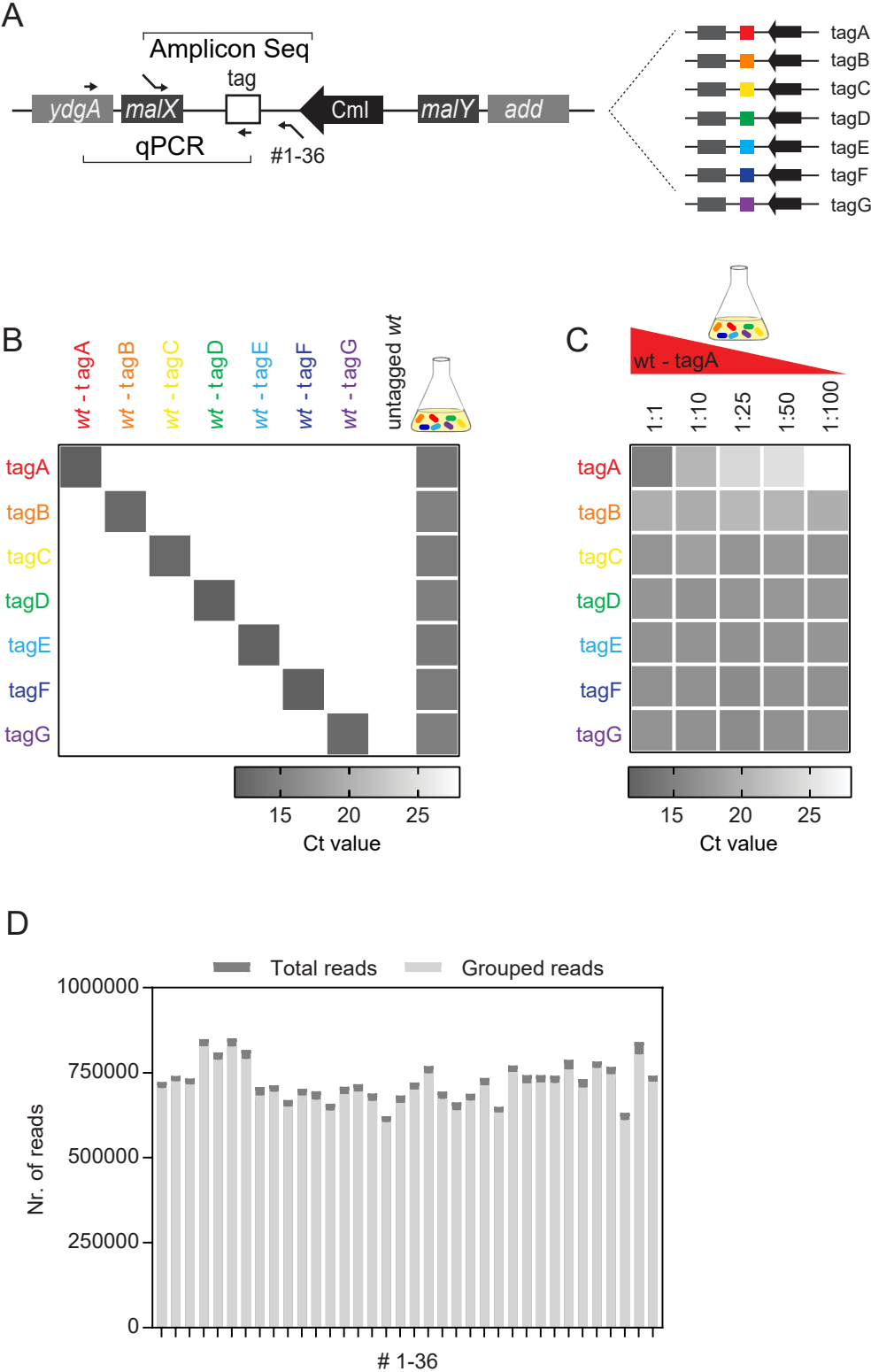

Supplement: FIG S1 [file mBio.00603-19-sf001.pdf]

Figure S2

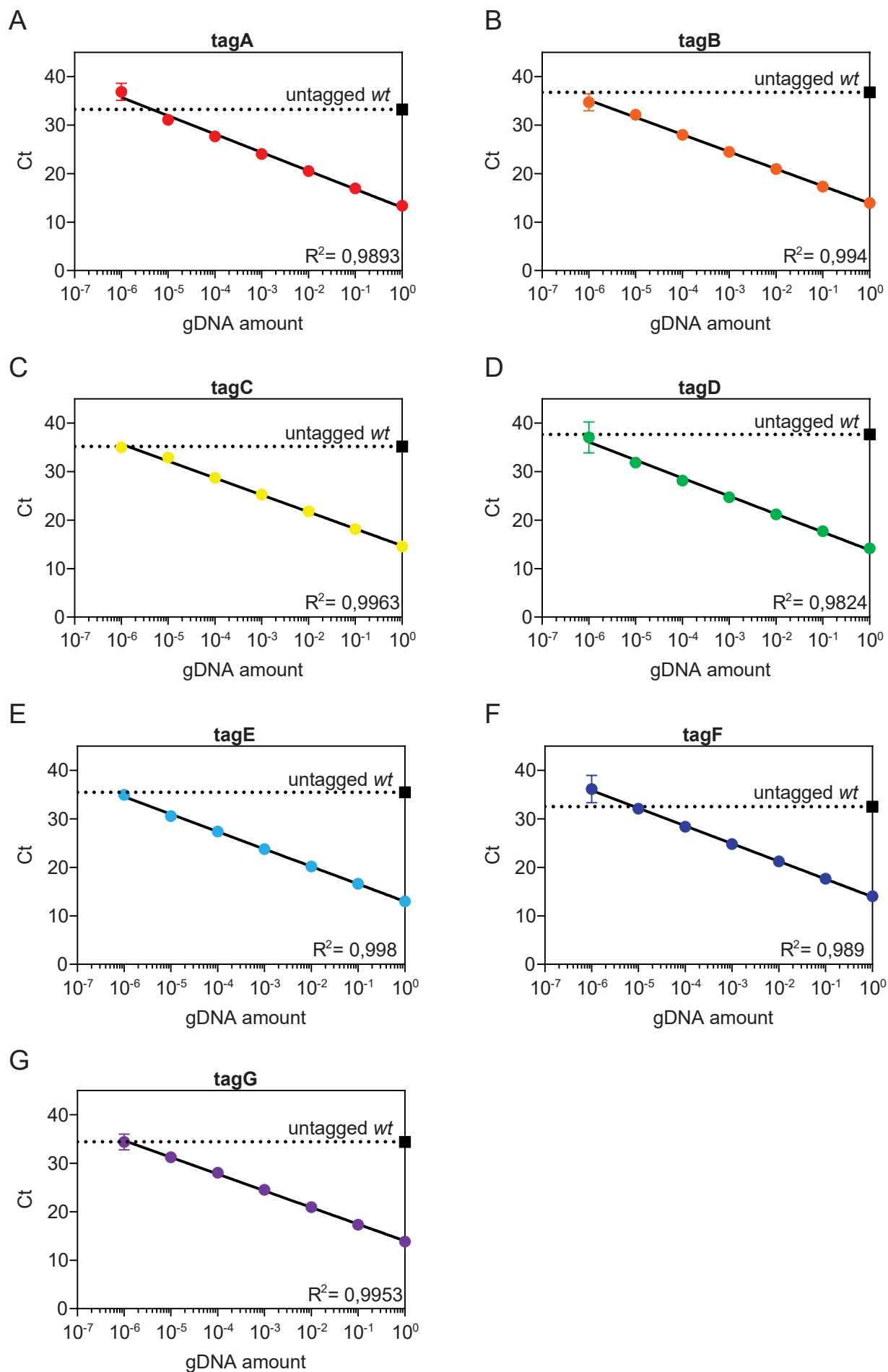

Supplement: FIG S2 [file mBio.00603-19-sf002.pdf]

Figure S3

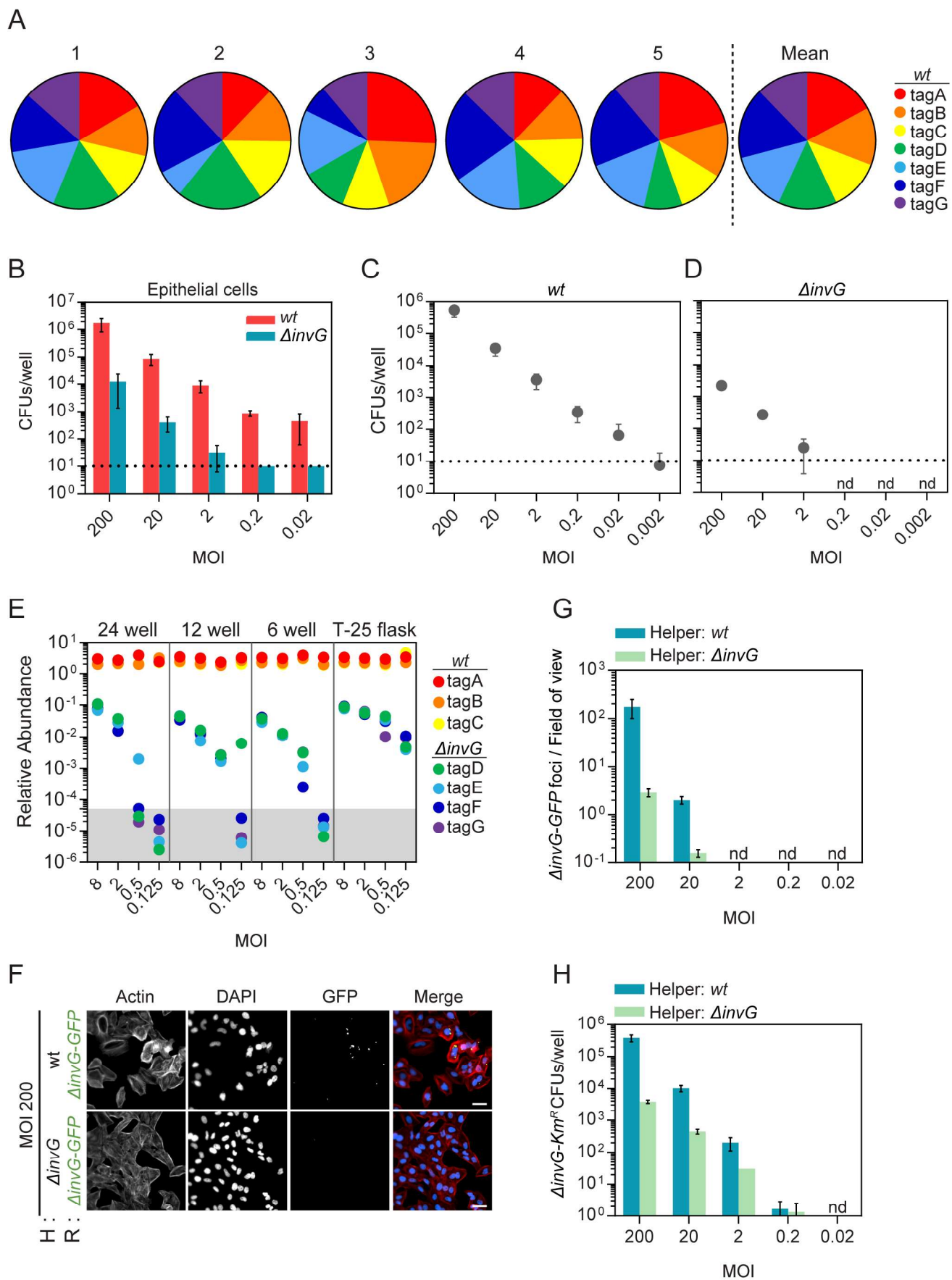

Supplement: FIG S3 [file mBio.00603-19-sf003.pdf]

Figure S4

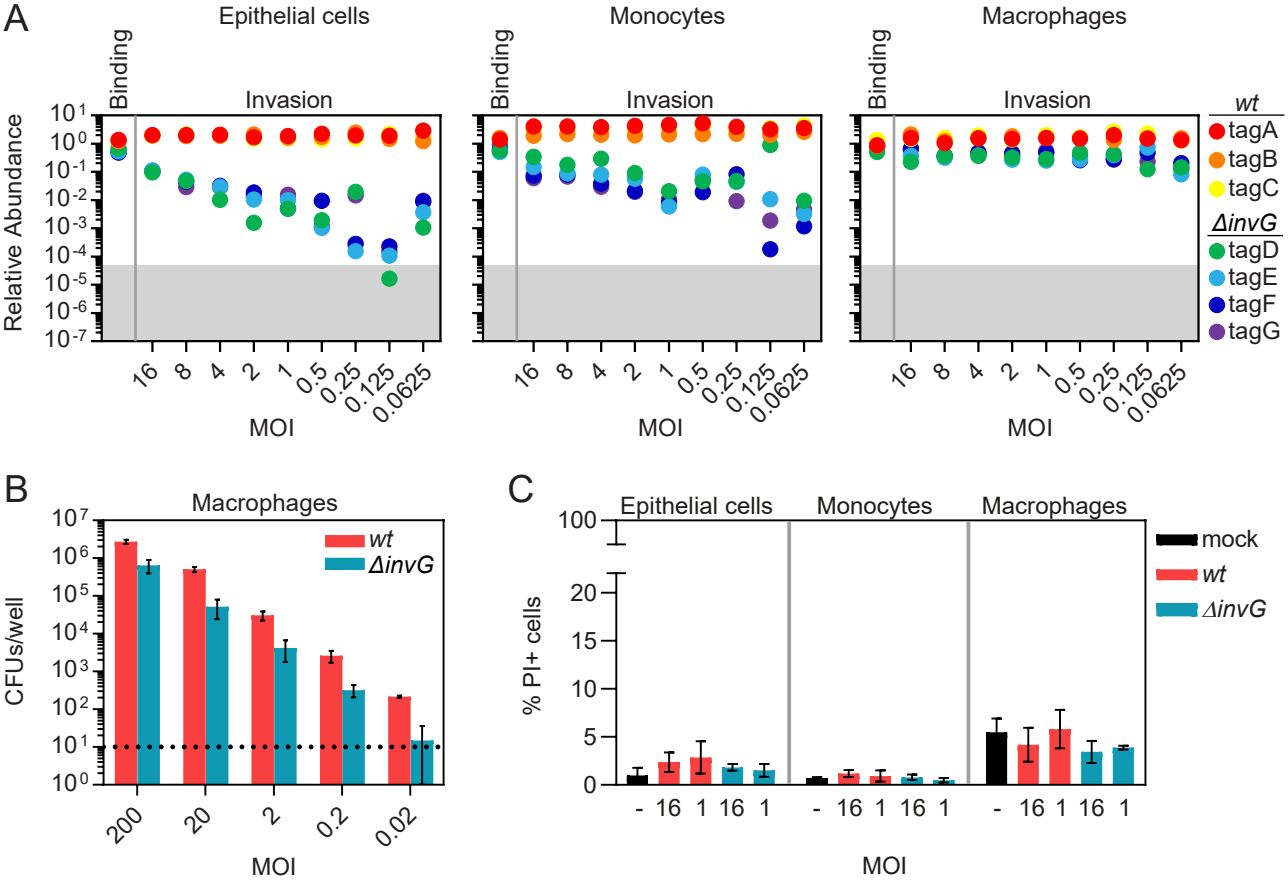

Supplement: FIG S4 [file mBio.00603-19-sf004.pdf]

Figure S5

A

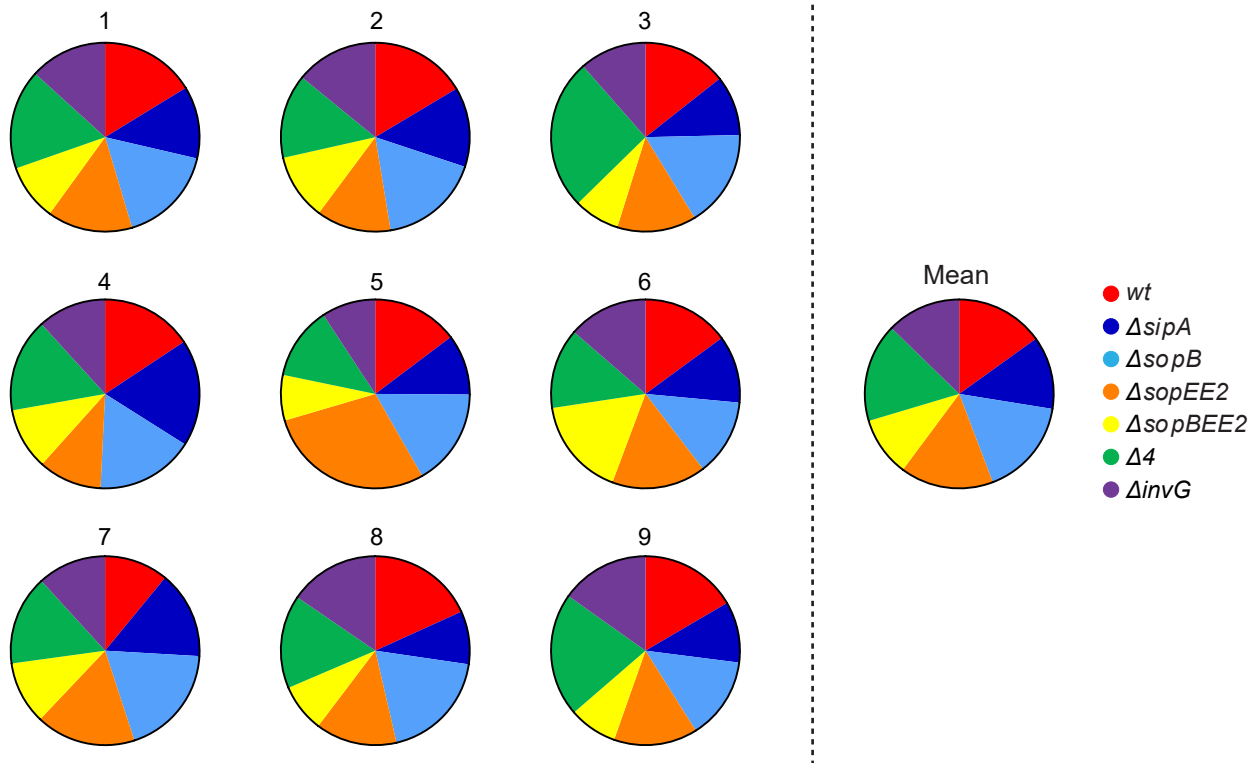

B

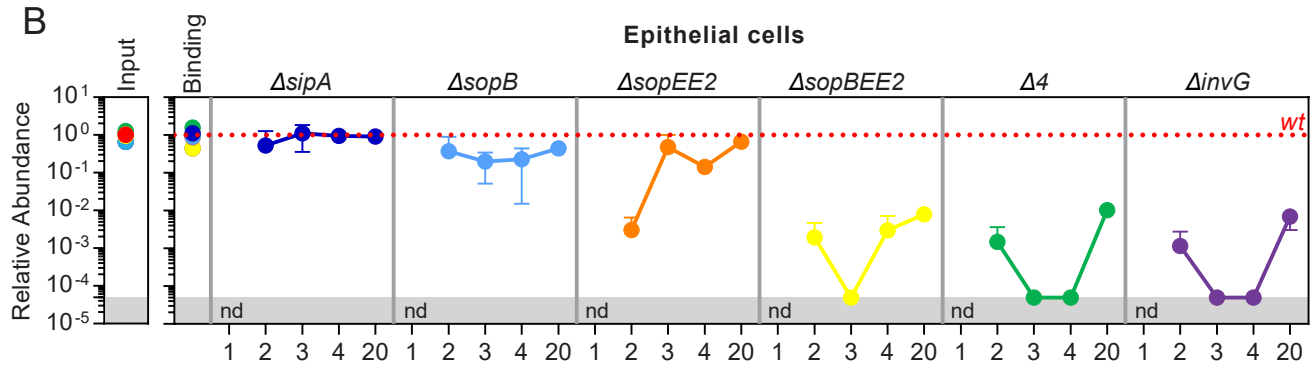

C

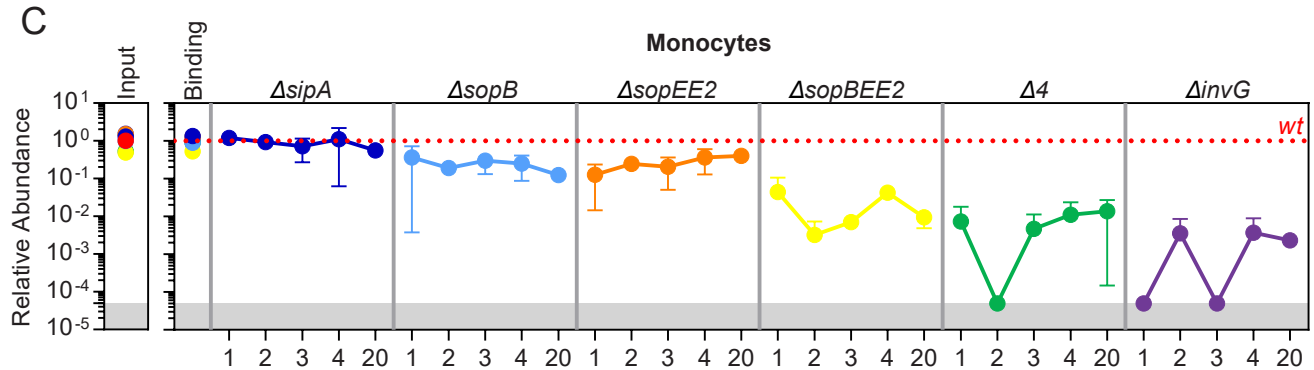

D

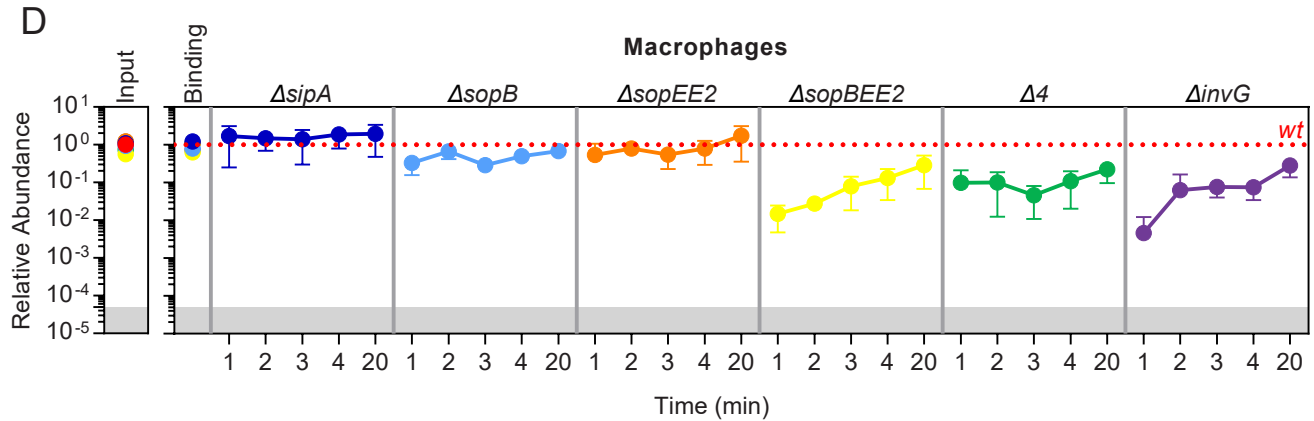

Supplement: FIG S5 [file mBio.00603-19-sf005.pdf]
