# Supplementary material for: Barcoded Consortium Infections Resolve Cell Type-Dependent Salmonella enterica Serovar Typhimurium Entry Mechanisms
Source: mBio. 2019 May 21;10(3):e00603-19. doi: 10.1128/mBio.00603-19 (PMC6529635; doi:10.1128/mBio.00603-19)
Supplement: TABLE S1 [file mBio.00603-19-st001.pdf]

Table S1. Chromosomal tag sequences and primers for qPCR and Amplicon Seq used in this study.

**Primer**	**Sequence 5'- 3'**	
Primers used for qPCR		Tag sequence detected
tagA	ACGACACCACTCCACACCTA	CGCGCGATAGGTGTGGAGTGGTGTCGTTGGGGGGGTTGTT
tagB	ACCCGCAATACCAACAACTC	GGGGAGTTGTTGGTATTGCGGGTGGTAGCTGGTGGGAGCT
tagC	ATCCCACACACTCGATCTCA	GGCGAGGGCGCGAGTGAGATCGAGTGTGTGGGATTGATAT
tagD	GCTAAAGACACCCCTCACTCA	AGCTGGAGCTCGAGAGTGAGTGAGGGGTGTCTTTAGCTAT
tagE	TCACCAGCCCACCCCCTCA	GGCGTGGGAGTGAGGGGGTGGGCTGGTGAGCGTTATAGTT
tagF	GCACTATCCAGCCCCATAAC	AGGGGTGGGGTTATGGGGCTGGATAGTGCGCGTGCTAGCT
tagG	ACAACCACCGATCACTCTCC	AGAGAGAGCTGGAGAGTGATCGGTGGTTGTGGTGGGAGTG
ydgA	GGCTGTCCGCAATGGGTC	(Common reverse primer for all tags)
Primers used for Amplicon Seq		
ionT F	CCTCTCTATGGGCAGTCGGTGATTGCAGGTAATTATCGGCCCG	
ionT barc_1 R	CCATCTCATCCCTGCGTGTCTCCGACTCAGCTAAGGTAACGATCTTGTGCAATGTAACATCAGAG	
ionT barc_2 R	CCATCTCATCCCTGCGTGTCTCCGACTCAGTAAGGAGAACGATCTTGTGCAATGTAACATCAGAG	
ionT barc_3 R	CCATCTCATCCCTGCGTGTCTCCGACTCAGAAGAGGATTCGATCTTGTGCAATGTAACATCAGAG	
ionT barc_4 R	CCATCTCATCCCTGCGTGTCTCCGACTCAGTACCAAGATCGATCTTGTGCAATGTAACATCAGAG	
ionT barc_5 R	CCATCTCATCCCTGCGTGTCTCCGACTCAGCAGAAGGAACGATCTTGTGCAATGTAACATCAGAG	
ionT barc_6 R	CCATCTCATCCCTGCGTGTCTCCGACTCAGCTGCAAGTTCGATCTTGTGCAATGTAACATCAGAG	
ionT barc_7 R	CCATCTCATCCCTGCGTGTCTCCGACTCAGTTCGTGATTCGATCTTGTGCAATGTAACATCAGAG	
ionT barc_8 R	CCATCTCATCCCTGCGTGTCTCCGACTCAGTTCCGATAACGATCTTGTGCAATGTAACATCAGAG	
ionT barc_9 R	CCATCTCATCCCTGCGTGTCTCCGACTCAGTGAGCGGAACGATCTTGTGCAATGTAACATCAGAG	
ionT barc_10 R	CCATCTCATCCCTGCGTGTCTCCGACTCAGCTGACCGAACGATCTTGTGCAATGTAACATCAGAG	
ionT barc_11 R	CCATCTCATCCCTGCGTGTCTCCGACTCAGTCCTCGAATCGATCTTGTGCAATGTAACATCAGAG	
ionT barc_12 R	CCATCTCATCCCTGCGTGTCTCCGACTCAGTAGGTGGTTCGATCTTGTGCAATGTAACATCAGAG	
ionT barc_13 R	CCATCTCATCCCTGCGTGTCTCCGACTCAGTCTAACGGACGATCTTGTGCAATGTAACATCAGAG	
ionT barc_14 R	CCATCTCATCCCTGCGTGTCTCCGACTCAGTTGGAGTGTCGATCTTGTGCAATGTAACATCAGAG	
ionT barc_15 R	CCATCTCATCCCTGCGTGTCTCCGACTCAGTCTAGAGGTCGATCTTGTGCAATGTAACATCAGAG	
ionT barc_16 R	CCATCTCATCCCTGCGTGTCTCCGACTCAGTCTGGATGACGATCTTGTGCAATGTAACATCAGAG	
ionT barc_17 R	CCATCTCATCCCTGCGTGTCTCCGACTCAGTCTATTCGTCGATCTTGTGCAATGTAACATCAGAG	
ionT barc_18 R	CCATCTCATCCCTGCGTGTCTCCGACTCAGAGGCAATTGCGATCTTGTGCAATGTAACATCAGAG	
ionT barc_19 R	CCATCTCATCCCTGCGTGTCTCCGACTCAGTTAGTCGGACGATCTTGTGCAATGTAACATCAGAG	
ionT barc_20 R	CCATCTCATCCCTGCGTGTCTCCGACTCAGCAGATCCATCGATCTTGTGCAATGTAACATCAGAG	
ionT barc_21 R	CCATCTCATCCCTGCGTGTCTCCGACTCAGTCGCAATTACGATCTTGTGCAATGTAACATCAGAG	
ionT barc_22 R	CCATCTCATCCCTGCGTGTCTCCGACTCAGTTCGAGACGCGATCTTGTGCAATGTAACATCAGAG	
ionT barc_23 R	CCATCTCATCCCTGCGTGTCTCCGACTCAGTGCCACGAACGATCTTGTGCAATGTAACATCAGAG	
ionT barc_24 R	CCATCTCATCCCTGCGTGTCTCCGACTCAGAACCTCATTCGATCTTGTGCAATGTAACATCAGAG	
ionT barc_25 R	CCATCTCATCCCTGCGTGTCTCCGACTCAGCCTGAGATACGATCTTGTGCAATGTAACATCAGAG	

ionT barc_26 R	CCATCTCATCCCTGCGTGTCTCCGACTCAGTTACAACCTCGATCTTGTGCAATGTAACATCAGAG
ionT barc_27 R	CCATCTCATCCCTGCGTGTCTCCGACTCAGAACCATCCGCGATCTTGTGCAATGTAACATCAGAG
ionT barc_28 R	CCATCTCATCCCTGCGTGTCTCCGACTCAGATCCGGAATCGATCTTGTGCAATGTAACATCAGAG
ionT barc_29 R	CCATCTCATCCCTGCGTGTCTCCGACTCAGTCGACCACTCGATCTTGTGCAATGTAACATCAGAG
ionT barc_30 R	CCATCTCATCCCTGCGTGTCTCCGACTCAGCGAGGTTATCGATCTTGTGCAATGTAACATCAGAG
ionT barc_31 R	CCATCTCATCCCTGCGTGTCTCCGACTCAGTCCAAGCTGCGATCTTGTGCAATGTAACATCAGAG
ionT barc_32 R	CCATCTCATCCCTGCGTGTCTCCGACTCAGTCTTACACACGATCTTGTGCAATGTAACATCAGAG
ionT barc_33 R	CCATCTCATCCCTGCGTGTCTCCGACTCAGTTCTCATTGAACGATCTTGTGCAATGTAACATCAGAG
ionT barc_34 R	CCATCTCATCCCTGCGTGTCTCCGACTCAGTCGCATCGTTCGATCTTGTGCAATGTAACATCAGAG
ionT barc_35 R	CCATCTCATCCCTGCGTGTCTCCGACTCAGTAAGCCATTGTCGATCTTGTGCAATGTAACATCAGAG
ionT barc_36 R	CCATCTCATCCCTGCGTGTCTCCGACTCAGAAGGAATCGTCGATCTTGTGCAATGTAACATCAGAG
