# Supplementary material for: Barcoded Consortium Infections Resolve Cell Type-Dependent Salmonella enterica Serovar Typhimurium Entry Mechanisms
Source: mBio. 2019 May 21;10(3):e00603-19. doi: 10.1128/mBio.00603-19 (PMC6529635; doi:10.1128/mBio.00603-19)
Supplement: TABLE S2 [file mBio.00603-19-st002.pdf]

Table S2. Bacterial strains used in this study.

Strain	Genotype	Reference (listed below)
S.Tm^{wt}	SL1344, *wt*	(1)
S.Tm^{wt}-tagA	SL1344, *wt*, WITS1-tag	(2)
S.Tm^{wt}-tagB	SL1344, *wt*, WITS2-tag	(2)
S.Tm^{wt}-tagC	SL1344, *wt*, WITS11-tag	(2)
S.Tm^{wt}-tagD	SL1344, *wt*, WITS13-tag	(2)
S.Tm^{wt}-tagE	SL1344, *wt*, WITS17-tag	(2)
S.Tm^{wt}-tagF	SL1344, *wt*, WITS19-tag	(2)
S.Tm^{wt}-tagG	SL1344, *wt*, WITS21-tag	(2)
S.Tm$^{\Delta invG}$-tagA	SL1344, *ΔinvG*, WITS1-tag	This study
S.Tm$^{\Delta invG}$-tagB	SL1344, *ΔinvG*, WITS2-tag	This study
S.Tm$^{\Delta invG}$-tagC	SL1344, *ΔinvG*, WITS11-tag	This study
S.Tm$^{\Delta invG}$-tagD	SL1344, *ΔinvG*, WITS13-tag	This study
S.Tm$^{\Delta invG}$-tagE	SL1344, *ΔinvG*, WITS17-tag	This study
S.Tm$^{\Delta invG}$-tagF	SL1344, *ΔinvG*, WITS19-tag	This study
S.Tm$^{\Delta invG}$-tagG	SL1344, *ΔinvG*, WITS21-tag	This study
S.Tm$^{\Delta sopEE2}$-tagB	SL1344, *ΔsopE, ΔsopE2*, WITS2-tag	This study
S.Tm$^{\Delta sopBEE2}$-tagC	SL1344, *ΔsopB, sopE::aphT, sopE2::tet*, WITS11-tag	This study
S.Tm$^{\Delta 4}$-tagD	SL1344, *ΔsipA, ΔsopB, sopE, sopE2*, WITS13-tag	This study
S.Tm$^{\Delta sopB}$-tagE	SL1344, *ΔsopB*, WITS17-tag	This study
S.Tm$^{\Delta sipA}$-tagF	SL1344, *ΔsipA*, WITS19-tag	This study
S.Tm^{wt}-GFP	SL1344, *wt*, p*rpsM*-GFPmut2	(3)
S.Tm$^{\Delta invG}$-*ssaG*GFP	SL1344, *ΔinvG*, p*ssaG*-GFPmut2	(4)
S.Tm$^{\Delta invG}$	SL1344, *ΔinvG*	(5)
S.Tm$^{\Delta invG}$-*Km*^{R}	SL1344, *ΔinvG*	This study
S.Tm$^{\Delta sipA}$	SL1344, *ΔsipA*	(6)
S.Tm$^{\Delta sopBEE2}$	SL1344, *ΔsopB, sopE::aphT, sopE2::tetR*	(7)
S.Tm$^{\Delta 4}$	SL1344, *ΔsipA, ΔsopB, sopE, sopE2*	(8)
S.Tm 14028-*ΔsopB* (C0835)	14028, *ΔsopB*	(9)
S.Tm 14028-*ΔsopE2* (C0977)	14028, *ΔsopE2*	(9)
S.Tm 14028-*ΔinvG* (C1793)	14028, *ΔinvG*	(9)

Table S2 references

1. Hoiseth SK, Stocker BA. Aromatic-dependent Salmonella typhimurium are non-virulent and effective as live vaccines. Nature. 1981;291(5812):238–9.

2. Grant AJ, Restif O, McKinley TJ, Sheppard M, Maskell DJ, Mastroeni P. Modelling within-host spatiotemporal dynamics of invasive bacterial disease. PLoS Biol. 2008;6(4):e74.

3. Stecher B, Hapfelmeier S, Mu C, Kremer M, Stallmach T, Hardt W, et al. Flagella and Chemotaxis Are Required for Efficient Induction of Salmonella enterica Serovar Typhimurium Colitis in Streptomycin-Pretreated Mice. Infect Immun. 2004;72(7):4138–50.

4. Hapfelmeier S, Stecher B, Barthel M, Kremer M, Müller AJ, Heikenwalder M, et al. The Salmonella Pathogenicity Island (SPI)-2 and SPI-1 type III secretion systems allow Salmonella serovar typhimurium to trigger colitis via MyD88-dependent and MyD88-independent mechanisms. J Immunol. 2005;174(1):1675–85.

5. Kaniga K, Bossio JC, Galán JE. The Salmonella typhimurium invasion genes invF and invG encode homologues of the AraC and PulD family of proteins. Mol Microbiol. 1994;13(4):555–68.

6. Hapfelmeier S, Ehrbar K, Stecher B, Barthel M, Kremer M, Hardt W-D. Role of the Salmonella Pathogenicity Island 1 effector proteins SipA, SopB, SopE, and SopE2 in Salmonella enterica subsecies 1 serovar Typhimurium colitis in streptomycin-pretreated mice. Infect Immun. 2004;72(2):795–809.

7. Mirold S, Ehrbar K, Weissmüller A, Prager R, Tschäpe H, Rüssmann H, et al. Salmonella host cell invasion emerged by acquisition of a mosaic of separate genetic elements , including Salmonella Pathogenicity Island 1 (SPI1), SPI5, and sopE2. J Bacteriol. 2001;183(7):2348–58.

8. Ehrbar K, Friebel A, Miller SI, Hardt W-D. Role of the Salmonella Pathogenicity Island 1 (SPI-1) Protein InvB in Type III Secretion of SopE and SopE2, Two Salmonella Effector Proteins Encoded Outside of SPI-1. J Bacteriol. 2003;185(23):6950–67.

9. Porwollik S, Santiviago CA, Cheng P, Long F, Desai P, Fredlund J, et al. Defined single-gene and multi-gene deletion mutant collections in Salmonella enterica sv typhimurium. PLoS One. 2014;9(7):e99820.
